# ‘When is a hotspot a good nanospot’ – review of analytical and hotspot-dominated surface enhanced Raman spectroscopy nanoplatforms

**DOI:** 10.1039/d3nr05332f

**Published:** 2024-01-17

**Authors:** Mike Hardy, Pola Goldberg Oppenheimer

**Affiliations:** a School of Chemical Engineering, College of Engineering and Physical Sciences, University of Birmingham B15 2TT UK GoldberP@bham.ac.uk; b Centre for Quantum Materials and Technologies, School of Mathematics and Physics, Queen's University Belfast Belfast BT7 1NN UK mhardy04@qub.ac.uk; c Healthcare Technologies Institute, Institute of Translational Medicine Birmingham B15 2TH UK

## Abstract

Substrate development in surface-enhanced Raman spectroscopy (SERS) continues to attract research interest. In order to determine performance metrics, researchers in foundational SERS studies use a variety of experimental means to characterize the nature of substrates. However, often this process would appear to be performed indiscriminately without consideration for the physical scale of the enhancement phenomena. Herein, we differentiate between SERS substrates whose primary enhancing structures are on the hundreds of nanometer scale (analytical SERS nanosubstrates) and those whose main mechanism derives from nanometric-sized gaps (hot-spot dominated SERS substrates), assessing the utility of various characterization methods for each substrate class. In this context, characterization approaches in white-light spectroscopy, electron beam methods, and scanning probe spectroscopies are reviewed. Tip-enhanced Raman spectroscopy, wavelength-scanned SERS studies, and the impact of surface hydrophobicity are also discussed. Conclusions are thus drawn on the applicability of each characterization technique regarding amenability for SERS experiments that have features at different length scales. For instance, while white light spectroscopy can provide an indication of the plasmon resonances associated with 10 s–100 s nm-scale structures, it may not reveal information about finer surface texturing on the true nm-scale, critical for SERS’ sensitivity, and in need of investigation *via* scanning probe techniques.

## Abstract 2

Substrate development in surface-enhanced Raman spectroscopy (SERS) continues to attract research interest.

## Graphical abstract

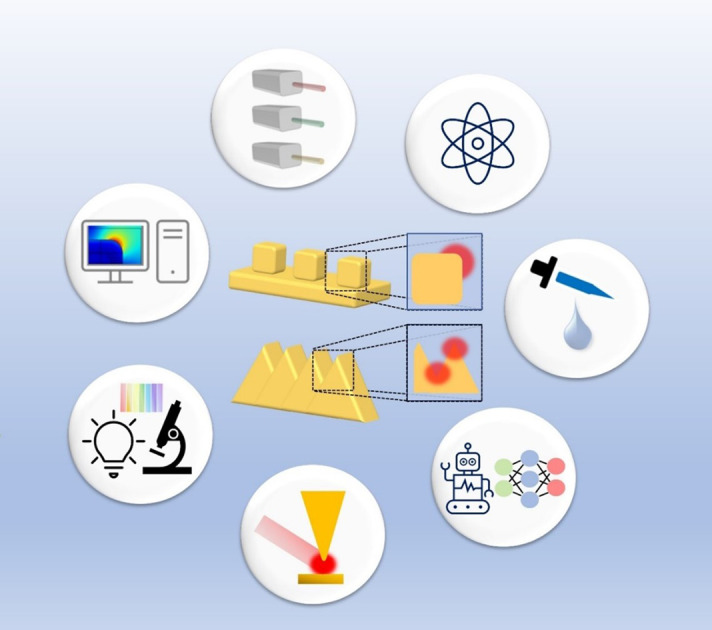

## 1. Introduction

Surface-enhanced Raman spectroscopy is a field that continues to move apace with ever more application-focused research into areas where trace detection is required.^1–28^ Concurrent with this, SERS researchers continue to explore new substrates, whether centered around new plasmonic materials^29–31^ or different nanostructure shapes,^32,33^ and naturally, a wide range of characterization methods are used. ‘Characterization’ is any process by which the properties of a substrate and subsequent performance metrics may be deduced. Natan (2005) emphasized the importance of metrics in SERS substrate evaluation, including those relating to sensitivity, uniformity and reproducibility, as well as longevity.^34^ Previously, focus has fallen on how the SERS enhancement factors have been calculated.^35^ Le Ru (2007) discussed differences in, for instance, substrate-averaged and single-molecule enhancement and highlighted variation in the Raman response from non-SERS reference molecules, some of which can have very different inherent Raman scattering intensities (Raman cross-sections). More recently, Bell (2020) has reviewed the different SERS substrate types, namely bottom-up and top-down, and the utility of various kinds of Raman reporter molecules.

Blum (2014) *et al.* performed an inter-laboratory tip-enhanced Raman spectroscopy (TERS) study,^36^ finding similar spectra between research groups irrespective of set-up used. The potential for further inter-laboratory enhanced Raman studies was discussed in ref. 16. Recently, Fornasaro (2020) *et al.* have engaged in a large pan-European SERS study, associated with the Raman4Clinics consortium, finding a standard error of performance metric as low as 12% or 13% between 785 nm measurements on the same silver nanopillar SERS substrates (Silemco ApS, Denmark) and same gold colloids (University of Padua – UNIPD, Italy).^37^ Likewise, Guo (2020) *et al.* have compared Raman spectrometer configurations across multiple labs encouraging more standard operating procedures, open data, and collaboration.^38^ These fundamental studies seek to determine the broad potential of Raman or SERS as a proper analytical tool. In the context of the translation of SERS to biomedical applications, it has been noted that variance across different SERS experiments constitutes a worthwhile discussion within the SERS community.^15^

Many different aspects of SERS theory and applications have been summarized recently in Langer (2019) *et al.*, a comprehensive review by a constellation of leading SERS researchers.^2^ While there is a clear awareness of the specific classes of SERS substrates *i.e.*, metal colloids/nanoparticles, lithographic approaches *etc*.,^32^ there may be an underappreciation of how optimal SERS characterization might be performed. This is important from an economical perspective: getting maximal information from minimal experimental endeavor. Moreover, appropriate SERS substrate characterization leads to more meaningful comparison of SERS substrates across different labs, still a problem in the SERS field,^17^ including benchmarking studies for novel platforms *versus* commercial substrates.^39,40^ Herein, we summarize the most prominent SERS characterization methods and comment on their applicability to different ‘types’ of SERS substrate. This is through the lens of the most striking division in SERS systems: whether the enhancement is judged to originate from larger features on the 10 s and 100 s nm-scale, which can be easily reproduced, or those more conventionally associated with the SERS phenomenon, with features on the nm-scale, SERS ‘hotspots’, but that cannot be reproduced or characterized so easily. We designate these broad descriptors as ‘analytical’ and ‘hotspot-dominated’ (HSD) SERS substrates.^41,42^

Feature size is thus important because it relates to reproducibility and sensitivity. Smaller features permit detection of the lowest analyte concentrations, but less reproducibly, there is less fabrication control on the truly nanometric-sized scale. Larger features, approximately on the 10 s nm-scale, concentrate the incident light less effectively, however in a more predictable way, thus providing better reproducibility across nanofabricated substrates, and indeed better uniformity of signal within any one substrate. This renders them a better option for analytical applications where precise quantification is required.

In SERS, the high plasmon-mediated electric fields surrounding nanofeatures couple to the incident photons, amplifying the signal. Critically, the process may be repeated after the photons have interacted with the molecule(s) of interest *i.e.*, post-Raman scattering. The enhancement in the de-excitation phase depends critically on the de-excitation wavelength of the Raman photons analyzed – this wavelength change is ‘the Raman shift’ – and the spectral width of the plasmon resonance *i.e.*, do the Raman-scattered photons overlap with the plasmonic excitation? The SERS enhancement mechanism can be understood then to be a highly non-linear effect, where potentially, the photons can be amplified in intensity pre- and post-interaction with the molecule, and hence the importance on high confinement of the plasmon for maximum sensitivity.

We note, the transition from a localized 10 s–100 s nm -confined surface plasmon-polariton oscillation, to one localized on the nm-scale, where it is considered a hotspot, is not well-defined. And thus, the difference between analytical and HSD SERS platforms can be blurry. From a physics perspective, plasmon-polaritons arising from features on the order of a few nanometres differ in that they are highly coupled modes, whereas plasmon-polaritons generated *via* larger surface features tend to be electromagnetically isolated. Moreover, truly nanometric features can also benefit from the lightning rod effect – the simple concentration of electric field lines around sharp features. Below nanometer separation, quantum tunneling effects come into play, which can lower local field intensities.

### History of SERS media

The electrochemical origin of SERS is well-known, where A. J. McQuillan first reported an anomalously large Raman signal of pyridine at a roughened silver electrode at a Faraday Discussion meeting in 1973,^43^ before publishing with Fleischmann and Hendra a year later.^44,45^ Unsurprisingly, then the initial research focus in SERS in the early years post-discovery concentrated on electrochemistry.^46^ Soon after, metal nanoparticles became prominent SERS media, being a quick, easy, and cost-effective way to promote hotspots and achieve high SERS enhancements. Nanoparticles (NPs) for SERS have continued to prove popular for these reasons, and different nanoparticle morphologies have been explored. Depositions of nanoparticles onto planar surfaces have also been studied, either *en masse* to form a ‘layer’ of nanoparticles on the surface,^47^ including printing on a paper substrate,^48^ or in a much more rarefied regime, namely, nanoparticle-on-mirror geometry where the focus is on a large electric field between the nanoparticle, a 2D material spacer layer, and a plasmonically active substrate.^49^ Hutter (2013) characterized the electric fields of a dielectric NP on a planar metal substrate,^50^ and previously Taylor (2011) controlled sub-nanometer gaps between AuNPs using macrocyclic molecules.^51^ Lee (2014) *et al.* have investigated swellable polymer media with interspersed metal nanoparticles for off-the-shelf SERS substrates.^52^ Core–shell nanoparticles have also garnered research attention, where a thin, inert outer shell has been employed to prevent unwanted adsorbative effects from analyte molecules,^53^ and potential chemical enhancements.^54^

Beyond electrochemically roughened surfaces and nanoparticles, there have been advances in easily produced, bottom-up SERS substrates that focus on high sensitivity, low cost, and scalability. Stenbaek-Schmidt (2012) introduced co-leaning silicon-based silver nanopillars for SERS that bend towards each other under the tensile forces of solvent molecules, and consequently form hotspots between abutting pillar caps.^40^ Šubr (2015) used oblique angle deposition (70–80°) to form rods leaning at high angles *via* a self-shadowing effect;^55^ the technique has more recently been reviewed by Chu (2017).^56^ Dawson and colleagues similarly employed angular deposition (15°) but with multi-walled carbon nanotubes (MW-CNTs), and only to apply a granular texture to the already formed structures.^57,58^ Goldberg-Oppenheimer and co-workers explored the optimization of erect CNTs for SERS purposes,^59^ and later, employed a capacitive set-up to induce instabilities in low viscosity polymer films, which produces upright structures for SERS.^60^ Nanopillars of various sorts have become a common bottom-up SERS platform and have been summarized by Oh (2016).^61^

In a seminal review, Banholzer (2008)^32^ reported on the progress of ‘rationally designed’ SERS substrates, including electromigrated or mechanical junctions,^62^ and anisotropic non-nanosphere-based engineered substrates.^63^ Top-down nanostructured SERS platforms have also come to prominence more recently, coinciding with progress into advanced nanofabrication techniques.^64^ These substrates are often lithographically produced, by photolithography, electron beam lithography, focused ion beam, or nanoimprint lithography, which confer different properties in terms of resolution, fidelity, and throughput.^65^ Highly ordered SERS substrates typically sacrifice a degree of SERS sensitivity by focusing on larger surface features that can provide better uniformity, spot-to-spot on one sample, and better reproducibility, batch-to-batch, across multiple samples. They are therefore of a ‘fundamentally different character’ to rougher, bottom-up SERS substrates,^41^ and are arguably a better solution for quantitative analyses. It is in this sense that hotspots in SERS are not always a ‘good spot’ for the analyst—the balance between required analytical sensitivity and uniformity/reproducibility should be considered. Commercial SERS substrates have also appeared, of both an analytical and hotspot-dominated characters, by companies such as Elliot Scientific Ltd (nanograins), Silmeco ApS (nanopillars), Nikalyte Ltd (nanoparticles on surface), SERSitive sp z.o.o. (nanoparticles on surface), and Renishaw Diagnostics Ltd with the discontinued Klarite inverted pyramidal SERS substrate. In recent years, progress in both bottom-up and top-down SERS substrates have been reviewed by Wang and Kong (2015), Jeon (2016) *et al.*, and López-Lorente (2021).^66–68^ New lithographic techniques could confer better trade-offs in terms of cost, throughput, size, and reproducibility for SERS, including electrohydrodynamic lithography, which uses instabilities in polymer films induced by a capacitive set-up, and stereolithography *aka* 3D printing, where lasers can be used to cure resin to precisely form smaller and smaller structures.

### Review outline

While the different characterization techniques in SERS are familiar to many, and a plethora of different kinds of SERS substrates exist, there is little discussion on the suitability of characterization techniques chosen, to the detriment of analyses. Herein, we provide overviews of characterization methods for SERS substrates commenting particularly on the suitability of each for the different types of SERS platforms: analytical and HSD substrates (see Table 1). We do not aim to comment on techniques that relate to the characterization of molecular species, or particular moieties, for example, use of resonant Raman arrangements *i.e.* SERRS, laser wavelength choice for fluorescence rejection, functionalized surfaces, or computational chemistry *e.g.* density functional theory, which have been discussed elsewhere.^69–73^ We note, however, that there is inevitably some overlap between analyte and substrate characterization in SERS studies. Moreover, we do not delve into the various ways that the SERS enhancement factor can be calculated, mathematically, or on what should be included *e.g.*, the increase in surface area by a particular nanogeometry. Different enhancement factor calculations have been provided in a publication by Le Ru (2007).^74^

**Table 1:** Summary of characterization techniques for SERS media. Applicability for analytical and hotspot dominated SERS platforms is indicated by color code: green = very suitable, amber = moderately suitable or suitable with caveat(s), red = (generally) unsuitable

	Analytic	HSD	Commentary
White light spectroscopy 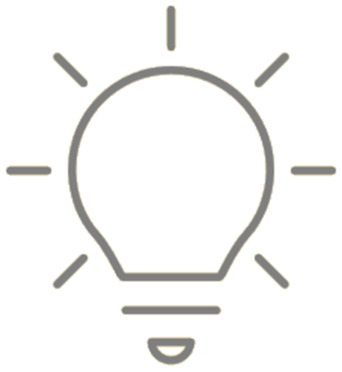	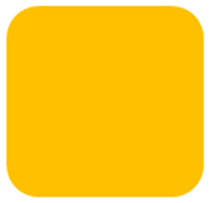	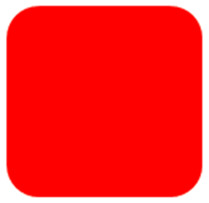	A simple and accessible characterization technique, but far-field optical spectra can be misleading: not necessarily indicative of near-field activity, especially for highly localized gap modes associated with HSD structures or EM-interacting nanostructures
			Optics used (*i.e.* NAs) in white light analysis and SERS experiments should be considered carefully for maximally meaningful comparison
Ellipsometry	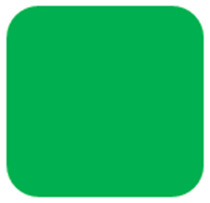	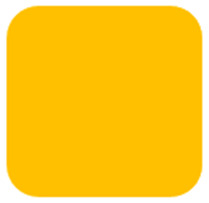	Ellipsometry can model layer composition and thickness, as well as surface roughness in some cases
Super-resolution microscopy	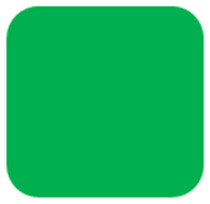	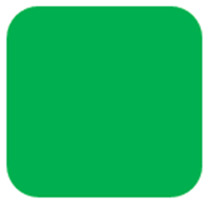	Super-resolution microscopy can be used to monitor nanometric hotspots by algorithmic assessment of optical data
Electron techniques 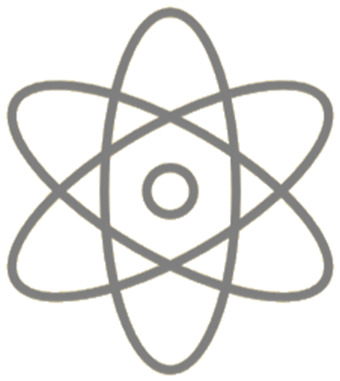			
SEM	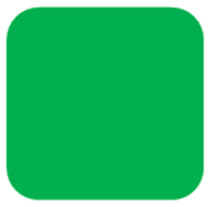	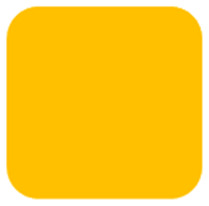	SEM, and image analysis, good for measurement of nanostructure period/disorder in large arrays and nm-size features. Precise identification of roughness parameters not possible. Multiple angles in SEM can be used to ascertain geometries
	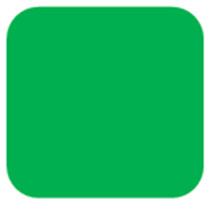	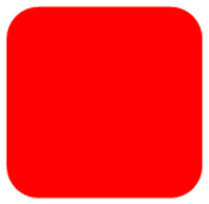	Low-current FIB can be used to obtain cross-sectional information in conjunction with SEM
	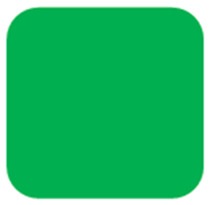	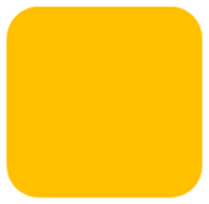	RISE can be used to correlate Raman and surface features
TEM	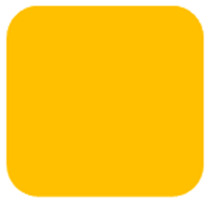	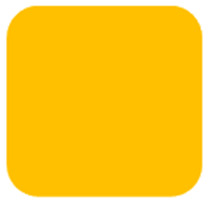	TEM is suitable for precise cross-section of nanostructures, as long as sample is robust enough. Sharp features in HSD substrates can be identified, and pm-scale atomic resolution can be interrogated to ensure adequate metal formation. Time-consuming procedure, required skilled users, and limited to thin slices
EELS	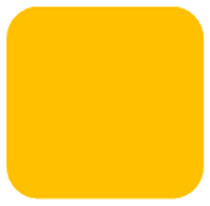	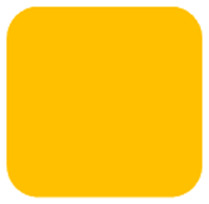	EELS offers possibility to analyse SERS hotspots and optically inactive ‘dark’ plasmons, but may excite other plasmons that would not be excited in plane wave optical experiments *e.g.* SERS. Like TEM, can damage sample and requires thin slices
PEEM/PEM	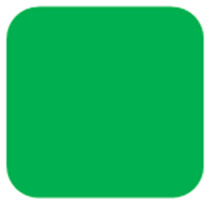	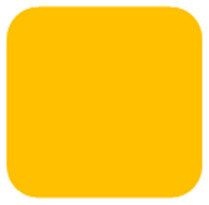	PEEM can use secondary electrons to image hotspots at 10 nm resolution. Can be overlaid with low-energy electron image to compare hotspots with surface topography
*Scanning probe microscopy*			
NSOM/SNOM 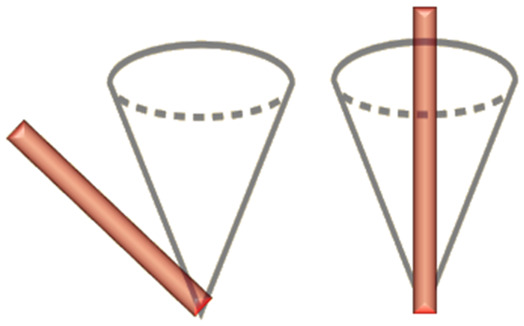	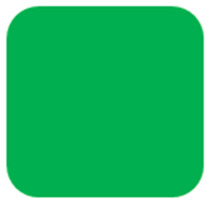	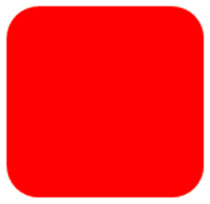	SNOM may be used to illuminate near-field plasmonic modes on the 10 nms-scale thus analyse larger plasmonic modes
AFM 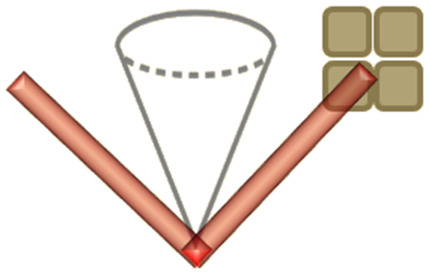	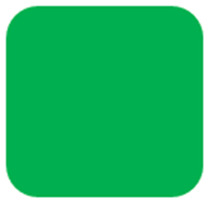	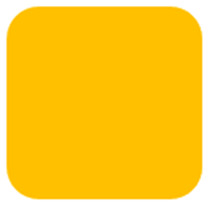	True 3D topographical assessment of 10 nms-scale structures, dependant on aspect ratio. Surface roughness can also be characterised but controller feedback settings must be finely tuned for accurate nanometric parameterisation
STM 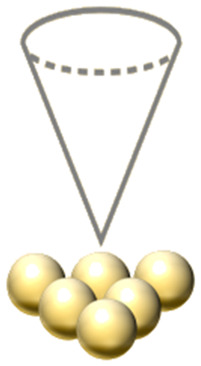	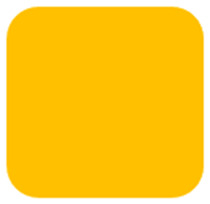	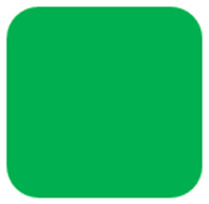	Similar to AFM, but faster and with atomic resolution; however, only 2D profiles possible. Impurities and film structure can be identified
TERS 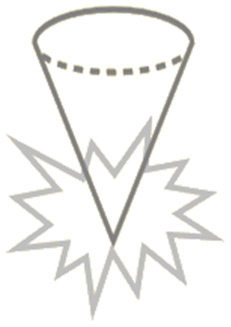	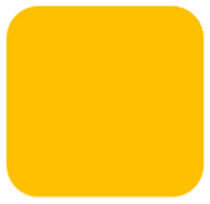	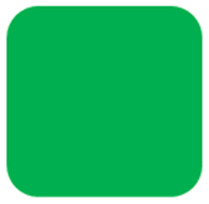	Plasmonic SPM tip brought near to SERS surface allowing precise control and analysis of hotspots between tip and nanostructures. Difficult experimental set-up and tip reproducibility is poor
WS-SERES 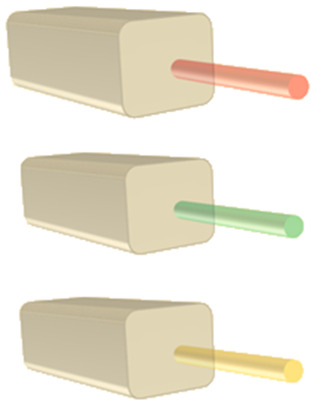	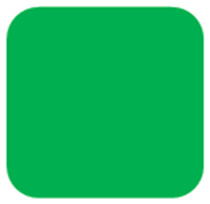	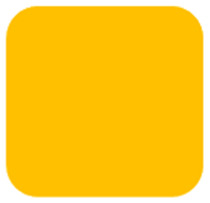	Using multiple laser wavelengths in experimental SERS. Allows ‘authentic’ assessment of plasmonic performance of SERS substrate across wavelength range. Can be used to properly evaluate near-field, far-field correlation. HSD substrates less likely to be ‘interesting’; wide range of plasmon energies excited and non-resonant lightning rod effect dominant. Time-consuming experiment with many lasers used
Hydrophobicity measurements 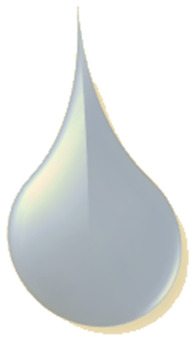	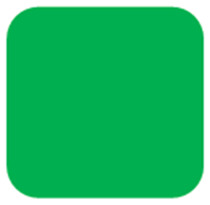	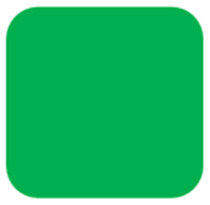	Initial contact angle measurements on a nanostructured surface can permit estimation of how analyte molecules will distribute across SERS-active area and interaction with plasmonic modes post-application. Theoretical understanding of wetting behaviour on nano/micro hierarchical structured systems is complex
Mathematical analysis 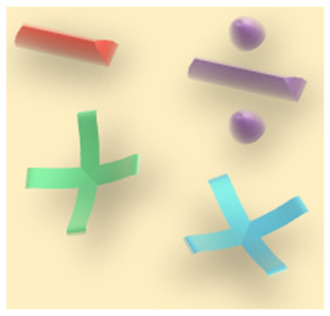	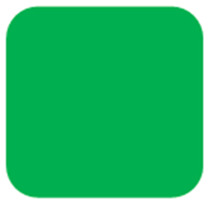	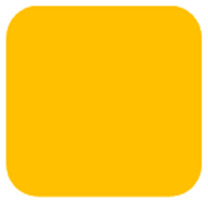	Analytical analysis can be performed within a broader plasmonic context *e.g.* SPP Bloch mode analysis in periodic SERS substrates. In a SERS-specific context, optomechanical models may contribute to better understanding nanometric-sized hotspots
Numerical methods 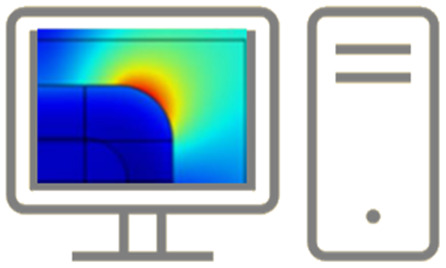	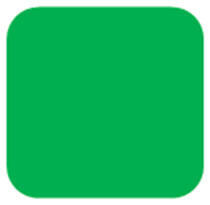	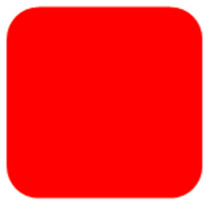	Numerical methods are good for simple SERS nanostructures: EM near-fields and thus SERS EF can be evaluated. Domain meshing must be appropriate. Extraction of reliable far-field spectra is difficult. Simulation of sharp features and nanometric roughness is often unrealistic. Quantum effects on sub-nm-scale are not accounted for; likewise electronic non-locality. Thermal effects, structural deformations, and microfluidics can also be simulated
Machine learning 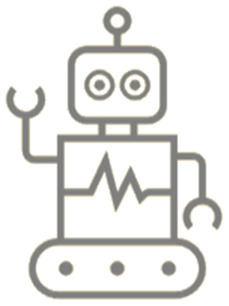	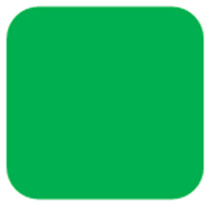	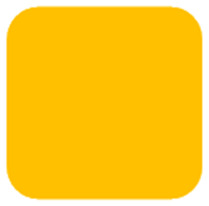	An emerging area in nanophotonics/plasmonics. Can be used to identify SERS hotspots for a specific geometry type based on prior (Raman-mapped) data, and could be used for SERS substrate design

Sections on electron microscopy (including electron energy loss spectroscopy), scanning probe microscopy, tip-enhanced Raman spectroscopy, and wavelength-scanned surface-enhanced Raman excitation spectroscopy where multiple laser wavelengths are used, are presented. Surface wetting can critically affect how analyte molecules are distributed on the surface and is thus discussed. First, however, we start with arguably the most common substrate characterization method in SERS studies: white light spectroscopy.

## 2. White light spectroscopy

Interrogation by simple reflection and transmission is often the first port of call in SERS substrate characterization. Here, the SERS substrate is examined with white light, typically at normal incidence. The attraction with white light spectroscopy is primarily due to the availability and interpretability – many laboratories have microscope systems and users are familiar with their use and data output. Perhaps underappreciated however is the impact of the exciting and collection optics. Any oscillator will have an inherent resonant position, a frequency at which it will be driven optimally, acquiring maximum amplitude (resonance). Alongside this, then, is also a range of slightly shifted frequencies, a ‘width’, where the oscillator is driven sub-optimally. This applies to the phenomenon of plasmon resonance where incident photons with a frequency (wavelength) act as the driving force for surface-bound electrons. However, the measured width of the resonance peak is also affected by system set-up. In excitation, a large numerical aperture (NA) objective lens provides many highly oblique incident photons that may stimulate different plasmonic surface modes to those at normal incidence. Contrariwise, a narrow, monochromatic light source used in the SERS evaluation will not use the full aperture of the focusing lens and thus experience a lower NA meaning very little angular spread of the photons incident on the SERS substrate. This discrepancy then can lead to a false impression of the width of plasmon resonances and that a laser wavelength and Raman-scattered wavelengths are optimally enhanced *via* overlap with the plasmon resonance wavelength, by the SERS effect when they are not. Similarly, if different optical systems are used for white light and Raman analysis, the NA used in collection is not always comparable and this is inadvisable for the same reason, different light cones (solid angles) will be collected by the two optical set-ups with the larger NA collecting more scattered light from the SERS substrate, also broadening the plasmon peak width. Where there is a strong reflective response from the substrate interface, dark-field measurements may be necessary where an aperture is inserted and the sample is illuminated with high-angle photons (only) and similarly, high-angle scattered light is collected (*e.g.*, >10° but depends on size and position of the aperture stop and the objective lens).

White light characterization in transmission is less common but can give more options than in reflection for collection optics, although reflection permits more faithful comparison with SERS measurements, which are usually in the backscattering (180°) configuration. In addition, transmission measurement is not possible for opaque substrates. Often, extinction is calculated *i.e.* −ln(transmission). The sample absorbance can be deduced *via* transmittance (% transmission) and reflectance (% reflection) measurements:

1 100% = %*T* + %*R* + %*A*

The interaction of photons with a medium can be defined in terms of transmittance (%*T*), reflectance (%*R*), and absorbance (%*A*).

Normally, off-normal measurements are not considered in SERS characterization studies; this may be unfortunate because higher angle measurements can be used to consider the effect of slight angular mismatch between laser and sample plane, which may occur due to experimental or fabrication imperfections. Optimal angle of incidence and collection in SERS are, in general, not considered, having been discussed early on in a Raman context by Greenler and Slager (1973)^75^ on a planar silver film, and more recently in a SERS context with spheres and cubes at a surface by Tian and colleagues.^16,76^

Confocal measurements are popular in Raman spectroscopy to provide compositional information at selected sample planes. The technique is typically of less interest to SERS research where the active nanostructures are at a surface. Recently, 3D SERS systems have emerged,^77–83^ conferring the benefit of potentially greater SERS signal with more regions of highly concentrated electric fields interrogated by a laser, for instance, the chestnut-like structures in the study of Huang (2017)^83^ (Fig. 2D), or silver-decorated ZnO nanorods in Tang (2012) (Fig. 2E).^81^ In addition to the usual problems with confocal microscopy relating to refractive effects when passing through layered media,^84^ it is unlikely that confocal measurements will yield much useful information about system hotspots: the best axial resolution achievable is ∼0.5 μm.

Ellipsometry is another white light measurement technique, and one that uses polarized light to calculate changes in phase and intensity at an approach angle of high incidence. Phase changes caused by the nanostructures can thus be recorded, and therefore discrimination may be made between this, and any phase change due to anisotropy in the Raman polarizability tensor of the analyte molecules [133]. While polarization effects are likely to be minor, they could have a sizeable effect on system sensitivity should polarizers be used in the optical set-up,^85^ or the polarization profile of a diffraction grating be sensitive to minor change. Ellipsometric evaluation entails the creation of a subsequent material model that estimates the composition of layer thicknesses, including the possible presence of impurities, which can be useful for SERS substrate characterization as the nature of the metal layer(s) are crucial in terms of the exact spectral profile of the plasmonic resonances supported. In addition, *via* adding a further artefactual (top) layer to the model, it can estimate surface roughness, although this so-called effective medium may be inaccurate for thin films.^86^ Similarly, ellipsometry also permits an assessment of the effect of an overlayer of analyte molecules (as is the case in the actual SERS experiment), which at a high enough surface coverage, could act as an additional dielectric layer that alters the spectral position of plasmonic modes, for instance, in the study of Ye (2012) *et al.* who notice spectral red-shifts of 70 nm in the *para*-mercaptoaniline Raman signature for a Fano heptamer system.^87^ Such an effect has also been discussed in the context of spectral shifts in gap plasmons by Baumberg and Sapienza in ref. 88.

### The near-field, far-field relationship

The electromagnetic (EM) near- and far-fields are those regions of space where the EM fields are nearest to the nanostructures, say within one wavelength dimension, and those far from the surface *i.e.* what is normally detected by a spectrometer and camera. The relationship between near-field and far-field properties of SERS substrates remains poorly understood.^89^ A selection of studies comparing the near-field and far-field in SERS have recently been tabulated in a wide-ranging SERS review by Pilot (2019) *et al.*^13^ That spectral position of features in the far-field optics, namely reflectance dips and transmittance peaks, should be aligned with the near-field SERS profile of a SERS substrate is, at first glance, not unreasonable, both phenomena are mediated by surface plasmon-polariton resonances,^90^ and indeed, many SERS reports make this assumption. Le Ru (2006) *et al.* highlighted the problem with equating maximal SERS performance with optical spectra noting that surface plasmon resonances could be either of more bulk character or more surface character. The former type provides optimal interaction between the external field and the substrate, showing a clear minimum in the far-field absorption, while the latter is much more localized and has little or no far-field signature but has a large impact on the SERS signal. Where interacting resonances are concerned, small changes *e.g.*, inter-structure spacing in a dimer system, can impact SERS but cause negligible far-field change.^91^

**Fig. 1:**
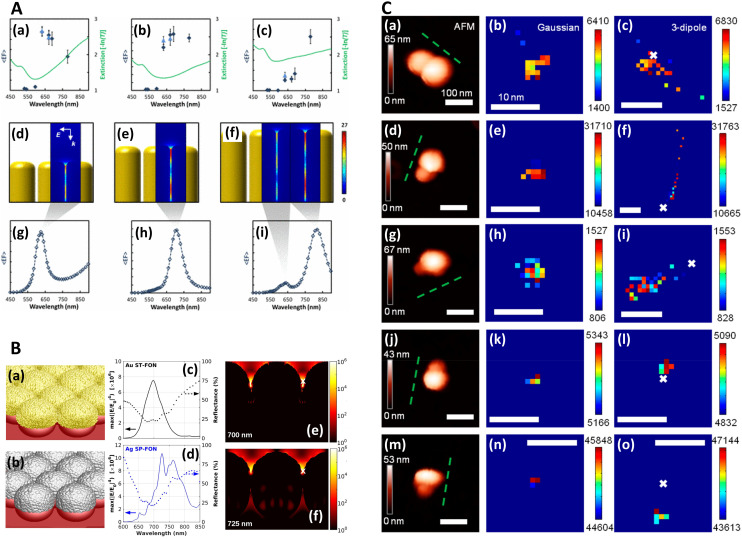
Near-field, far-field studies in SERS. A. Experimental and modeled SERS enhancement factor, demonstrating the effect of a nanopillar cavity mode on SERS. (a)–(c) Graphs comparing the measured (raster-averaged) enhancement factor 〈EF〉 and optical extinction [−ln(*T*)] at normal incidence for nanorods with an average diameter of 48 nm; array period of 64 nm; and average lengths of (a) 85, (b) 125, and (c) 160 nm. The green lines indicate the optical extinction. Black-diamond and blue-triangle data points indicate the 〈EF〉 of the 915 cm^−1^ band of CV and the 775 cm^−1^ band of R6G, respectively. (d)–(f) Schematics of the modeled nanorod structures with 3 nm separation; array period of 64 nm; and lengths of (d) 85, (e) 125, and (f) 160 nm, overlaid with plots of local field on excitation of the third harmonic of the cavity. The fifth harmonic is also shown in (f) for the longest nanorods. (g)–(i) Graphs displaying the EF predicted by the finite-element method modeling for the nanorods shown in (d)–(f). The hollow diamond data points indicate the predicted enhancement factor for an imaginary 850 cm^−1^ Raman band. All enhancement factors are plotted at the excitation wavelength. Reprinted with permission from Doherty (2013) © American Physical Society.^90^ B. Finite-difference time-domain (FDTD) calculations for ‘spun’ and ‘stationary’ films over nanospheres nanostructured SERS substrates (SP-FONs and ST-FONs). Modeled geometries used in the FDTD simulations for the (a) Au SP-FON and (b) Ag ST-FON, respectively. Reflectance spectra (dotted lines) and fourth power of the near field (|*E*/*E*_0_|^4^, solid line) calculated within the gap (white mark in panels (e) and (f)) for the (c) Au SP-FON and (d) Ag ST-FON, respectively. Spatial distribution of |*E*/*E*_0_|^4^ around the (e) Au SP-FON (*λ* = 700 nm) and (f) Ag ST-FON (*λ* = 725 nm), respectively. Reprinted with permission from Kurouski (2017) © American Chemical Society.^89^ C. Superlocalization Surface-Enhanced Raman Scattering Microscopy. Atomic force microscopy (AFM) images and spatial intensity maps that indicate the average intensity of all centroids located within 1 nm bins. (Left column) AFM image with a three-dipole *γ* axis-fit (*x*–*y* plane) estimate indicated by the dashed line, (center column) spatial intensity map using a 2D Gaussian model, and (right column) spatial intensity map using a three-dipole model. A white X indicates the average position of the 2D Gaussian centroid. Panels (a)–(i) show single molecule SERS examples, with multi-molecule SERS examples in (j)–(o). Reprinted with permission from Titus (2013) © American Chemical Society.^97^

In a multiwavelength study, Doherty (2013)^90^*et al.* demonstrated that the optical properties of gold nanorods showed a mismatch with calculated experimental and theoretical SERS enhancement factors (EFs) (Fig. 1A). Here, regions where the optical extinction is high (−ln[transmission]) do not necessarily correspond to the highest SERS enhancement. The EF calculation used is2
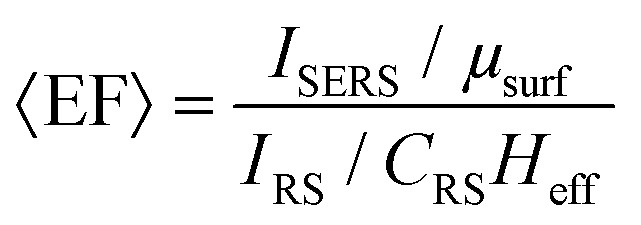
Typical experimental SERS EF calculation relating unenhanced Raman measurements to SERS measurement with plasmonic structures present. Where *I*_SERS_ is the SERS signal intensity, *I*_RS_ is the unenhanced Raman signal intensity, *μ*_surf_ is the density of molecules on the SERS surface, *C*_RS_ is the concentration of the moelcules without the SERS surface, and *H*_eff_ is the effective height of the laser, a measure of the volume of analyte probed by the laser (typically in a cuvette). 〈EF〉 represents an average enhanced Raman measurement.

For their numerical simulations, the authors use the finite element method and the equation:3
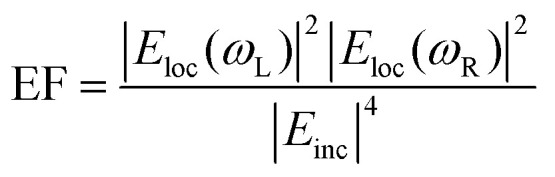
Typical numerical SERS EF calculation relating the simulated electric field without any nanostructures present to the simulated near-fields with the plasmonic model structures introduced. Here *E*_LOC_ represents the local electric field concentrated by the nanostructures, at frequencies (wavelengths) for the laser (*ω*_L_) and Raman band (*ω*_R_), and *E*_INC_, the incident field without any nanostructures.

For a hypothetical Raman peak approximating the de-excitation wavelength of dye molecules. These calculations are typical of how experimental and theoretical SERS EFs are calculated. However, a plethora of different SERS enhancement factor calculations exist, which still can prove a problem for cross comparison of EFs in the SERS literature. For example, whether a substrate-average measurement is considered, or the EF evaluated at a more localized region (or even the ‘hottest’ spot!), or whether the areal increase caused by the nanostructures, over a truly planar substrate, is considered.

The EF mismatch between the experimental and numerical simulations occurs because the SERS enhancement largely depends on the concentrated electric fields in a localized standing wave plasmonic cavity mode between adjacent pillars, and less so on the longitudinal (long axis) and transverse (short axis) modes that readily appear in far-field optics. Proximal pillar arrangements are not routinely observed in the study but are concluded to contribute disproportionately to the SERS signal.^90^ Likewise, Kleinman (2013) *et al.* observed a lack of correlation between the localized surface plasmon resonance (LSPR) spectral position and maximal SERS wavelength in multiparticle systems *via* computational modeling and deployment of multiple excitation wavelengths (see section on wavelength-scanned SERS: Fig. 6B). The authors cite the need to account for retardation effects—modification of the plasmonic response as the exciting wavelength of the light becomes comparable to nanostructure size—and the excitation of optically inaccessible plasmon-polariton modes mediated by Raman dipole re-radiation. Crucially, this means that not only does a discrepancy exist between SERS and far-field optics for electromagnetically interacting nanostructures as part of an ensemble, but that this mismatch can also be present with isolated structures.^92^

Similarly, Kurouski (2017) *et al.* demonstrated that the textured nature of SERS nanostructures could significantly affect the agreement between near- and far-field observations (Fig. 1B, also see Fig. 6C). In their films over nanospheres (FON) study, larger ‘blob’ nanofeatures on the FON surface increase radiative damping and thus induce spectral red-shifts in the far-field.^89^ There may be other reasons for discrepancies between SERS and optics, for instance, Banbury (2019a) *et al.* conveys that a 3D SERS co-block polymer design can show optical properties more indicative of the 3D structure than plasmonic interactions *i.e.* a photonic crystal/metamaterial-like signature in the far-field spectra.^79^

### Super-resolution spectroscopy

In order to image nanometric hotspots with white light, Willets and co-workers,^93–97^ as well as Cang (2011) *et al.*,^98^ introduced the procedure of super-resolution imaging *via* the use of a 2D Gaussian model to overcome the diffraction limit. Titus and Willets (2013) showed that molecular position determination could be improved *via* a three-dipole point spread function model (Fig. 1C).^99^ In a subsequent review, Willets (2014) discusses the simple Gaussian approach in the context of analyzing SERS hotspots, summarizing that although the model is often unphysical in its description of hotspots, typically a multi-dipole emission problem, that a 2D Gaussian fit tends to be a good approximation in most cases.^100^4
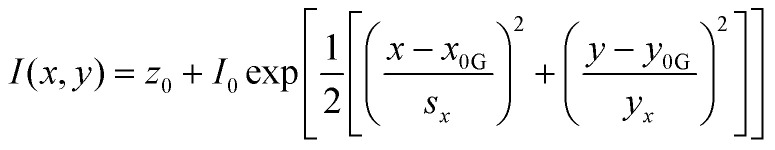
A 2D Gaussian fit that can be used to achieve super-resolution in white light microscopy, where *I* is the intensity of the diffraction-limited emitter, *z*_0_ is the background, *I*_0_ is the maximum intensity, *x*_0G_ and *y*_0G_ are the positions of maximum intensity, and *s*_0G_ and *s*_0G_, the related Gaussian standard deviations in *x* and *y*.

Thus, there is a range of issues to consider when characterizing SERS substrates *via* optical spectroscopy. Where the SERS substrate has μm-scale features, the incident and collection optics might be considered carefully, with high-angle photons resulting from a large NA exciting different plasmonic modes (or at least the expected modes less efficiently), and where a large NA in collection, might collect many scattered photons that obscure the signature of the plasmon-polaritons in the optical spectra. This is less likely to be of importance for more nanometrically sized and erratically shaped surface features, or larger bottom-up SERS microstructures that already have a range of orientations respective to the exciting field. Further, the potential lack of far-field response of small features associated with HSD substrates should be remembered; ordinary white light imaging may not be optimal for many nanometrically sized, rough-featured bottom-up substrates, and instead, super-resolution imaging can be used to elucidate surface hotspots.

## 3. Electron microscopy and spectroscopy

### Scanning electron microscopy and micrographic analyses

Another staple of SERS substrate characterization is scanning electron microscopy (SEM), which uses a focused beam of electrons to produce an image of a sample surface using secondary or reflected (back-scattered) electrons. SEM requires little sample preparation, provides resolution on the order of 1 nm, and compositional analysis is also possible by measuring characteristic X-rays (energy-dispersive X-ray spectroscopy, EDX). Micrographs can be analyzed post-acquisition with simple measurement software to determine geometrical dimensions, as well as pitch in an ordered nanostructured array. Where nanostructure disorder is present, the mean and range of inter-structure spacings can similarly be evaluated. This can also be characterized across the whole SERS-active area *via* use of Voronoi tessellation (or similarly Delaunay triangulation), which seeks to define areal cells with the property that every part of such a cell is less than or equally proximal to a point amongst a prescribed point set, here, set at nanostructure centers. The approach has recently found use in the characterization of natural photonic crystals.^101,102^ In a SERS context, it may be useful where either the characteristics of nanostructures (geometry, pitch) can vary significantly over the SERS-active region^103^ and inter-structure electromagnetic independence or interaction is important.^104^ While precise characterization of truly nanometric surface asperities is difficult, SEM can elucidate areas of larger-scale roughness, for instance, regions around nanostructure bases where metallization is less conformal, roughened arborescent caps on erect structures,^40^ or grainy surface formations,^105^ and which may transform an analytical SERS platform into one of more HSD-type.

SEM micrographs can also be used to calculate or verify packing density (and precise areal increase due to SERS nanostructures), although more nanostructures do not always constitute greater SERS EFs. For instance, Wei (2016) *et al.* describe a non-monotonic increase in SERS EF as the distance between cylindrical nanostructures is decreased, where modest near-field coupling (fill factor 0.2–0.35) can delocalize electromagnetic fields.^104^

One particularly useful and novel technique is that of combined SEM and Raman analysis (RISE), where a Raman intensity map is overlaid on top of a concurrently acquired SEM micrograph (Fig. 3).^106–109^ This permits a microscale evaluation of where on the substrate the predominant SERS activity presides and is therefore helpful for assessing whether a substrate is of more analytical or HSD character. A subsequent histogram analysis of the Raman intensity in the 2D map of pixels can provide a qualitative view of the type of SERS substrate *i.e.*, hotspot intensity and distribution, and this can be quantified with derived descriptive statistics metrics. Such an analysis assumes a uniform Raman analyte distribution, which can be problematic in SERS studies across a mm area,^110^ but is less so on the μm-scale, where molecular uniformity can be assumed unless phenomena relating to molecular clumping or preferential deposition induced by the presence of the nanostructures is apparent. In some cases, meaningful assessment of a 3D SERS system may be possible, as by Štolcová (2015) *et al.* who presented 3D hotspots in silver-coated glass fibers as a function of depth, which align with cross-over points between the structures. While similar resolution constraints exist as in (normal) confocal microscopy, the overlaid SEM micrograph adds meaning to the acquired data.^106,111^ RISE is part of a trend of increasing correlative nanotechnology characterization approaches.^112^

**Fig. 2:**
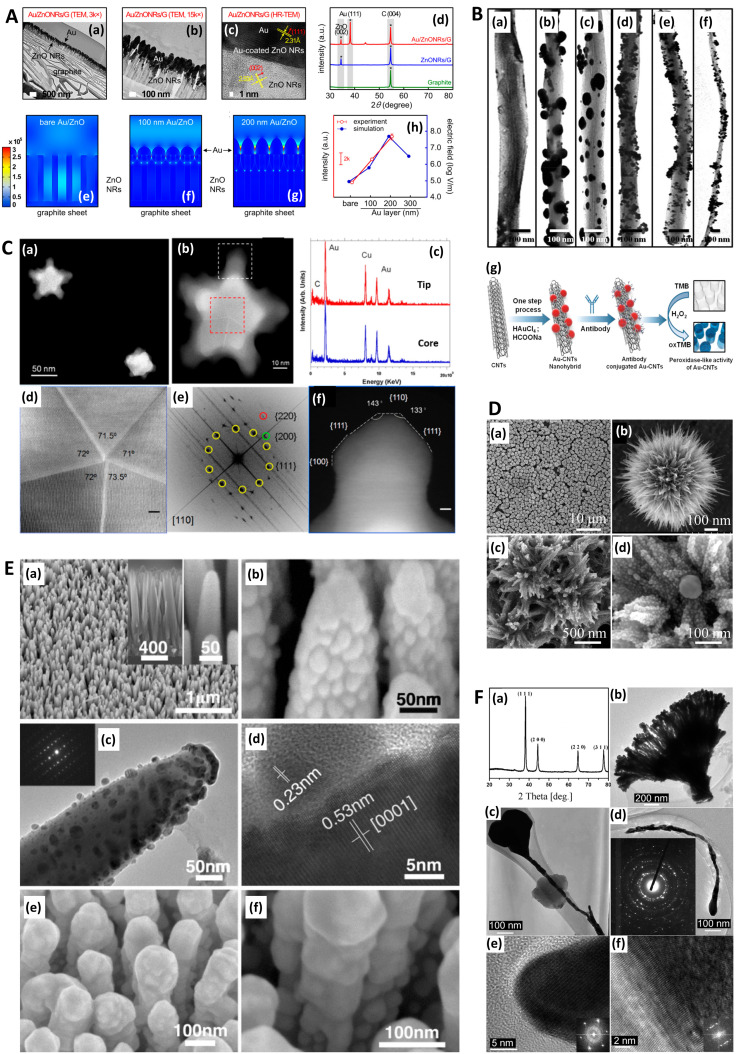
Electron microscopies in SERS. A. Gold-coated zinc oxide nanorods (ZnO NRs) for SERS: experimental characterization and modeling. (a and b) Transmission electron microscopy (TEM) and (c) high-resolution transmission electron microscopy (HR-TEM) images of Au/ZnO NRs/G and (d) X-ray diffraction patterns of graphite, ZnO NRs/G, and Au/ZnO NRs/G. (e)–(g) Computational results of FEM models approximated from (e) bare Au as well as (f) 100 nm thick and (g) 200 nm thick Au layers coated onto ZnO NRs with heights of 400 nm on graphite sheets. (h) Similarity of experimental findings (Raman intensity) and FEM computational findings (maximum electric field) with three models. G = graphite. FEM = finite element method. Reprinted with permission from Kim (2017) © American Chemical Society.^128^ B. TEM images of Au–CNT nanohybrids. (a) Carbon nanotubes (CNTs); (b) Au–CNT nanohybrids with ∼60 nm-sized AuNPs; (c) Au–CNT nanohybrids with ∼40 nm-sized Au NPs; (d) Au–CNT nanohybrids with ∼25 nm-sized Au NPs; (e) Au–CNT nanohybrids with ∼10 nm-sized Au NPs; (f) Au–CNT nanohybrids over a large-scale area (∼3 mm). (g) Schematic illustration of the one-step preparation of Au–CNT nanohybrids using HCOONa and chloroauric acid (HAuCl_4_). The antibody was conjugated with the Au–CNT nanohybrids through amide bonding, and proof of peroxidase-like activity based on colorimetric detection of virus deposited on 96-well plates was established. In the absence of Au–CNT nanohybrids, the 3,3′,5,5′-tetramethylbenzidine (TMB)–H_2_O_2_ mixed solution was colorless. In the presence of Au–CNT nanohybrids, the oxidized TMB (oxTMB)–H_2_O_2_ solution produced a strong blue color. Reprinted with permission from Ahmed (2016) © Elsevier.^124^ C. TEM characterization of nanoshurikens for SERS. (a) Low-magnification, high-angle annular dark-field (HAADF) scanning transmission electron microscopy (STEM) image of gold nanoshurikens (AuNShs) differently oriented with respect to a carbon support film, respectively. (b) Medium-magnification HAADF-STEM image of one of these AuNShs, consisting of a 5-fold star structure composed of a decahedral core and five tips. (c) X-ray energy-dispersive spectroscopy (EDS) spectra recorded at the core and at the tip, marked by the red and white squares, respectively. Carbon, copper (both elements come from the TEM grid), and gold are visible in the EDS spectra. (d) HAADF-(HR)STEM micrograph of the core of the nanoparticle showing its decahedral structure. The 5-fold symmetry and the multiple twinning domains are clearly evidenced. (e) Fast Fourier transform (FFT) obtained from the red square marked area of (b) (equivalent to (c)). For the sake of clarity, the color of this FFT is inverted. The superimposed contribution of each of the crystals composing the decahedral core, rotated with respect to each other, of the {111}, {200}, and {220} planes, is marked with yellow, green, and red, respectively. (f) HAADF image of the tip marked by a white square in (b). HR = high resolution. Reprinted with permission from Morla-Folch (2014) © American Chemical Society.^150^ D. Chestnut-like nanostructures for SERS. (a and b) Representative SEM images of formed 3D chestnut-like non-stoichiometric tungsten (sub)oxide (WO_2.72_) nanostructures, (c and d) SEM images of AgNPs decorated WO_2.72_ nanochestnuts. Reprinted with permission from Huang (2017) © American Chemical Society.^83^ E. Characterization of silver-coated zinc oxide nanorods (ZnO NRs) for SERS. (a) SEM image of the wafer-scale arrays of cone-shaped zinc oxide nanorods. Left inset: side view; right inset: enlarged image of the tapered ZnO NRs. (b) SEM and (c) TEM image of the ZnO NRs after an Ag-sputtering for 135 seconds. The inset is the selected-area electron diffraction pattern taken from the ZnO NR. (d) A lattice-resolved TEM image of the ZnO NR and AgNP adjacent surface. (e) SEM image of ZnO NRs with large Ag spheres on their tops. (f) SEM image of small AgNPs on the side surface of the ZnO NRs after an Ag-sputtering for 12 minutes. Reprinted with permission from Tang (2012) © John Wiley and Sons.^81^ F. Characterization of dendritic gold nanostructures (DGN) for SERS. (a) X-ray diffraction (XRD) pattern and (b–f) TEM images of the 3D-DGNs: (b) a whole view of a 3D-DGN; (c and d) high-magnification TEM views of two typical well-dispersed nanowires, inset of d is a selected-area electron diffraction pattern of the gold nanowire; HR-TEM images of head (e) and tail (f) of the nanowire, insets of (e) and (f) are the corresponding Fourier transform patterns. Reprinted with permission from Lu (2007) © American Chemical Society.^77^

**Fig. 3:**
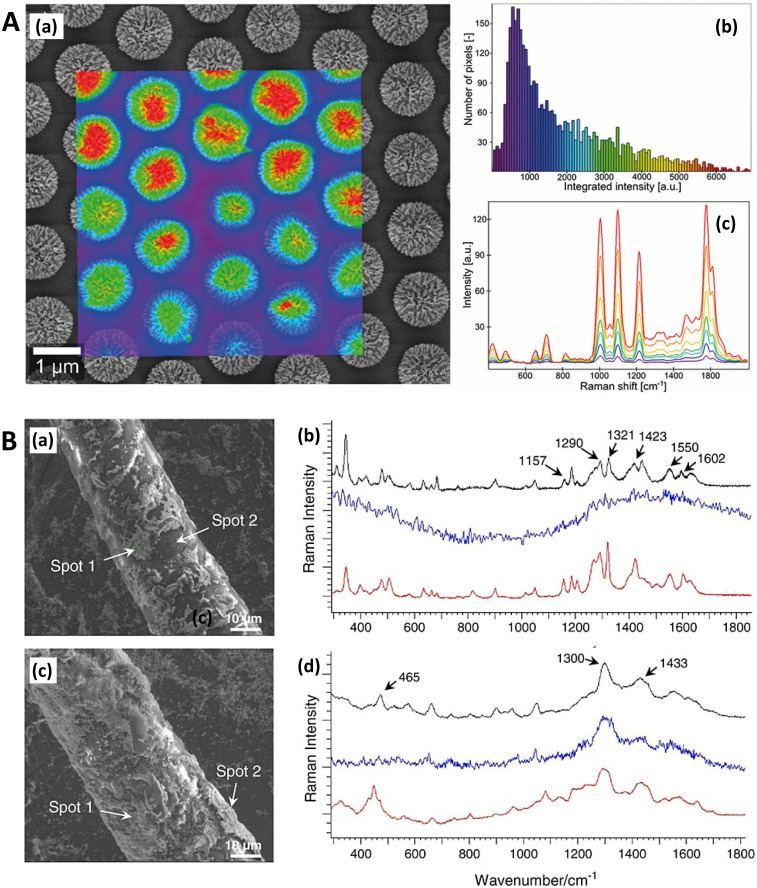
Raman-SEM Correlative Imaging in SERS-active nanostructures. A. Raman-SEM correlative imaging (RISE) of nanocoral array. (a) SEM image (mixture of secondary and backscattered electron signal) overlaid with a false-color SERS map of the identical area, colors from violet to red correspond to different values of SERS intensity (from low to high, respectively) calculated as the area of 1215 cm^−1^ peak; (b) histogram of the integrated SERS intensities; (c) corresponding SERS spectra of 4-mercaptopyridine. Reprinted with permission from Štolcová (2015) © John Wiley and Sons.^106^ B. RISE of dyed fibers for SERS (a) SEM image of an alpaca fiber dyed with cochineal. (b) Stack of the SERS spectra measured in the SEM using the Structural and Chemical Analyzer (SCA) (Renishaw) on the spot 1 (black line) and 2 (blue line) shown in (a) and SERS spectrum of the reference sample of carminic acid obtained in the confocal optical microscope (red line). (c) SEM image of a wool fiber dyed with chay root. (d) Stack of the SERS spectra measured in the SEM using SCA on spot 1 (black line), and 2 (blue line) shown in (c) and SERS spectrum of the reference sample of alizarin obtained using the confocal optical microscope (red line). Reprinted with permission from Prikhodko (2015) © John Wiley and Sons.^108^

Focused ion beam (FIB) may be used to obtain a cross-section of SERS nanostructures and hence verify geometrical parameters. This includes overlaid plasmonically active metal layers, which may not be even or conformal, and hence affect plasmon-polariton modes supported and resulting SERS EF. Geometrical verification might be of special importance where the fabrication procedure has an inherent morphological variability, for instance, nanoimprint lithography (NIL), or where there is change to the metalized layer over time *i.e.*, migration of metal. In many cases, cross-sectioning is unnecessary: there is strong alignment between the prescribed fabrication parameters and realized structures, or else a range of parameters is present in the final structures, and this is acceptable. And often, the precise parameters of final structures are clear from SEM. FIB is cumbersome to perform requiring specialized users, and thus, is probably only to be performed sparingly, even where it is of value.

Moreover, the use of aggressive characterization methods assumes robustness of the sample, and this is unlikely where, for instance, NIL is used and a low thermal conductivity polymer is employed as the base material. The problem can be resolved somewhat with additional thermally conductive overcoated materials, preferably ones with variable density to provide suitable contrast in a subsequent SEM micrograph showing the cross-section (*e.g.* amorphous carbon: ∼1 W m^−1^ K^−1^@293 K; gold or tungsten: ∼100 W m^−1^ K^−1^@293 K). Additionally, a fine 10 s pA mill can refine the cross-section once the initial trench is exposed. Nevertheless, it is difficult to ascertain whether small structure deformations have occurred. FIB is also used to prepare thin slices for analysis with transmitted electrons, in transmission electron microscopy.

### Transmission electron microscopy (TEM)

Transmission electron microscopy (TEM) is another electron microscopy technique but where the electrons are passed through the sample, typically a sub-150 nm cross-sectional slice (lamella). TEM can provide precise structural details with atomic resolution (10 s pm scale) but is aggressive thus requiring a robust sample. In an early study by Kruszewski (1994) *et al.*, the authors used TEM to determine the optimum grain size for SERS on roughened silver electrodes. They found that grain sizes with dimensions of 10 s nm-scale, achieved within a few oxidation–reduction cycles (ORCs), gave the largest SERS intensity. Subsequent ORCs produced grain features beyond 100 nms and lower SERS signals, the extent to which depended on the oxidation potential applied.^113^ However, most SERS-TEM studies examine the precise geometrical details of metal nanoparticles,^114–121^ including bimetallic core–shell structures,^122,123^ and different-sized gold nanoparticles adhered to carbon nanotubes for the SERS detection of influenza A (H3N2) virus (Fig. 2B).^124^ Recently, Lenzi (2021) *et al.* have used correlated SERS and TEM to discern the SERS signal per individual plasmonic nanoparticle and have produced a freely available app (‘SERSTEM’).^121^

Some substrate-based SERS characterization studies with TEM also exist. Soundiraraju (2017) *et al.* study the platelet-like μm-scale structure of Ti_2_N, from the MXene family of 2D materials, noting that optimum SERS is achieved when Ti_2_N is integrated with the fibrous structure of a paper substrate.^125^ Khlebtsov (2015) *et al.* use TEM images for high-fidelity numerical models of the near-field profiles in gold island films, estimating a 30 nm thickness in all simulations.^126^ Meanwhile, Lu (2017) *et al.* explore dendritic gold SERS structures, using TEM to discern the elongated nm-scale morphology as well as the structure of the gold *via* electron diffraction images, a 2D projection of the reciprocal lattice (Fig. 2F).^77^ This can be compared with a Fourier transform of the known crystal lattice, as in the TEM-SERS study of Dar (2012) *et al.*^127^ Fourier analysis can also be used to visualize nanostructure order by mapping spatial features to the frequency domain as in the ESI of ref. 103. This could be useful to detect a slight aperiodicity on a sample-sized scale should more overt indicators be insufficient *e.g.* visual iridescence. In a study into the classification of human aqueous humors, Kim (2017) *et al.* characterize a gold-coated ZnO nanopillar SERS nanoarchitecture on graphite with high resolution TEM, determining a ZnO layer-to-layer spacing of 2.59 Å corresponding to the hexagonal plane (002) of wurtzite (*P*63*mc* space group). This was corroborated with X-ray diffraction analysis (XRD) and suggested that the structures are well-formed on the graphite sheet without contamination (Fig. 2A).^128^ One of the problems with substrate analysis for TEM is the laborious preparation sequence where a slice, typically <100 nm, must be cut. Moreover, preparation *via* FIB can result in implanted ions redeposited with amorphized material, which can then alter the plasmonic response.

Nevertheless, TEM can be useful in both analytical and HSD substrate evaluations. For analytical designs, inter-nanostructure gaps can be accurately measured, and the crystallographic structure of the substrate and overcoated plasmonic layers can inform on the presence of imperfections that are likely to affect the dielectric properties of the metal *i.e.* having a perturbative effect on plasmon-polariton formation. For HSD substrates, the positions and precise geometry of sharp features dictating the SERS enhancement can be determined. TEM and SEM can be combined to take advantage of the merits of both techniques in the form of scanning transmission electron microscopy (STEM). This is also often a set-up for the related technique of electron energy loss spectroscopy.

### Electron energy loss spectroscopy

Electron energy loss spectroscopy (EELS) measures the distribution of energy changes from a transmitted beam of electrons of known energy and can therefore elucidate plasmonic excitations (alongside other excitations such as phonons, and electronic inter- and intra-band transitions). Willets (2012) gave an overview of EELS in the characterization of plasmonic nanoparticles,^129^ and the mathematical treatment of optical excitations in electron microscopy was expounded by Garcia de Abajo (2010) in a seminal review.^130^ The electronic probe means that not only transverse plasmons can be excited, but also ‘pure’ plasmonic modes, which are longitudinal excitations.^131^ Unlike the transverse hybridized electron-light modes associated with SERS, true charge density oscillations are not excited by light and thus not of interest in SERS research. Transverse bulk plasmon-polaritons also exist but are also not of much importance to SERS, merely akin to a slightly modified photon as it travels through a medium. Within plasmonics, EELS is probably most frequently associated with the early theoretical plasmon work of Ritchie (1957),^132^ experimental investigations by Powell and Swan (1959, 1960),^133,134^ and further theoretical validation by Stern and Ferrell (1960).^135^

In a modern SERS context, EELS is not a common characterization technique. An early investigation compared the SERS and EELS spectra for benzene with some interesting discrepancy. While benzene's different bonding orientations on a silver surface were clearly seen in EELS spectra, it was less apparent in the SERS spectra, suggesting that surface bond strength is much less applicable to the latter.^136^ Another notable study investigating chemical enhancement on Cu(100) and Cu(111) surfaces was conducted by Kambhampati (1998) *et al.* and^137^ the same authors have also examined charge transfer resonances in SERS with EELS.^138^ From a SERS substrate characterization perspective, the cardinal attraction of EELS is that it may be able to measure plasmonic gap (interfacial) modes with high spatial (<1 nm) and energy resolutions (0.1–0.3 eV), which are not possible in the far-field,^139^ but necessary for SERS hotspot evaluation.^140^

**Fig. 4:**
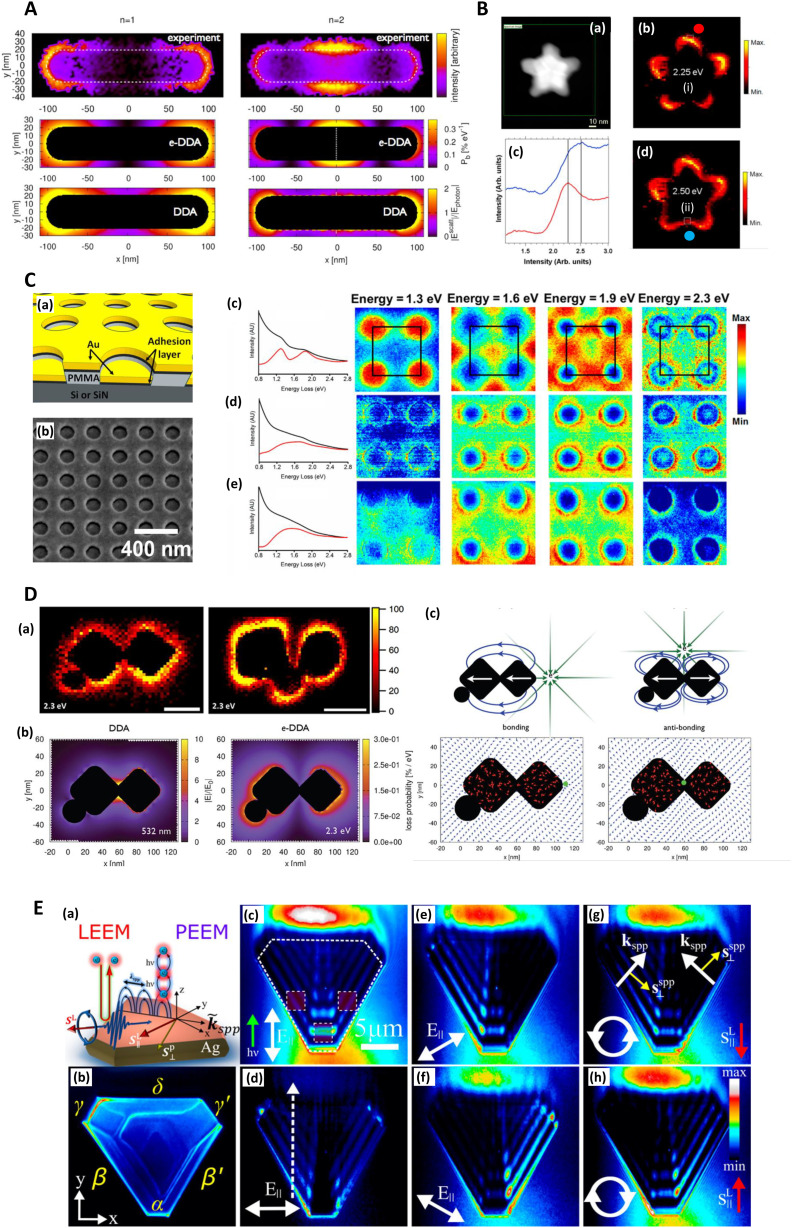
Electron energy loss (EELS) and photoemission electron microscopy (PEEM) and SERS. A. EELS loss-probability maps of the two lowest-lying longitudinal plasmon modes of a silver nanorod supported on an amorphous SiN_*x*_ substrate. *n* = 1 (1.50 eV) and *n* = 2 (2.61 eV) correspond to the first bright and dark plasmon modes of the monomer. The upper two panels display the experimentally measured loss-probability maps, adapted from Guiton (2011),^233^ while the middle two panels display the same observable computed *via* an electron-driven discrete dipole approximation (e-DDA). Each map indicates where in space the incident electron is likely to deposit the fraction *ħω* of its initial 0.1 MeV kinetic energy into a multipolar plasmon mode. The white dotted line in the middle right panel indicates the spatial location of the node of the first dark plasmon mode of the rod monomer. The lower two panels display the magnitudes of the corresponding electric fields scattered from the rod after excitation by a plane wave, computed *via* the DDA; these near-field magnitudes are taken in ratio to the magnitude of the incident plane wave, *E*_photon_. For the *n* = 1 mode, the incident field's direction of propagation (electric polarization) is normal (parallel) to the long axis of the rod. While for the *n* = 2 mode, the incident field propagation and polarization directions lie in the plane of the long axis of the rod and its normal but are tilted by ±45° with respect to the normal. This arrangement allows for light to couple into a mode of the rod that is dark under normal incidence. To symmetrize the *n* = 2 scattered electric field, we average together ±45-polarizations (see Guiton 2011^233^). It is clear that the loss-probability maps (upper four panels) and the photonic local density of states (see Fussell 2005^234^), which is related to the scattered electric field magnitude (Novotny 2006^235^) (bottom two panels), are not simply related to each other in this case (see Hohenester 2009;^236^ García de Abajo 2008^237^). Reprinted with permission from Bigelow (2012) © American Chemical Society & Guiton (2011) © American Chemical Society.^156,233^ B. EELS characterization of nanoshurikens for SERS. (a) High-angle annular dark-field (HAADF) scanning transmission electron microscopy (STEM) of a gold nanoshurken (AuNSh). EELS spectrum-imaging has been recorded in the square area marked in green. (b) and (d) Intensity maps extracted from the EELS spectrum-imaging after removing the zero-loss peak. The intensity maps show the spatial distribution of the two excited LSPR modes of the AuNSh, noted as (i) and (ii). (c) EELS spectra (each of them corresponds to the sum of 9 spectra) extracted from the EELS spectrum-imaging in the areas marked in each of the intensity maps (square regions) and marked as red dot (red line in (c)) and blue dot (blue line in (c)). Reprinted with permission from Morla-Folch (2014) © American Chemical Society.^150^ C. EELS of nanodisks for SERS with focus on plasmonic damping effect of adhesion layers. (a) Schematic diagram of the structures investigated. The thicknesses are 30 nm for Au, 2 nm for the adhesion layer, and ∼100 nm for polymethyl methacrylate (PMMA). (b) SEM of the actual structure. The sample is tilted to illustrate better the vertical offset between the gold nanodisks and the surrounding thin film. (c–e) Left: Spectra summed over the whole 500 × 500 nm region of interest before (black) and after (red) background subtraction of samples with (c) no adhesion layer, (d) 2 nm Ti, and (e) 2 nm Cr. Right: Normalized STEM-EELS energy slices generated from the three samples at energies 1.3, 1.6, 1.9, and 2.3 eV. Each slice is generated from a ± 0.05 eV range of the listed energy and normalized by total detector counts. The four nanodisks shown here are within a larger array, with the array unit cell outlined in black. In order of increasing energy, resonances appear in the sample with no adhesion layer (c) at unit cell corners, face centers, edge centers, and corners again. Samples with (d) Ti and (e) Cr adhesion layers do not show such localized high-intensity features. Reprinted with permission from Madsen (2017) © American Chemical Society.^139^ D. Spatially resolved EEL maps for single-molecule SERS (SMSERS)-active trimers. (a) Images (loss energy of 2.3 eV) have been normalized to the zero-loss peak (ZLP). A complete EEL spectrum is obtained for every pixel in the region of interest (defined by the annular dark field; however, focus is on the loss energy of 2.3 eV as this corresponds to the energy of the Raman laser (532 nm, 2.3 eV) used in the SMSERS experiment). While it is assumed that the largest electromagnetic enhancement is obtained at the gap region, no localization of the EEL intensity is observed in the gaps. Scale bars are 50 nm (left) and 100 nm (right). (b) Comparison of the calculated electric near-field magnitude obtained from plane-wave excitation (left) with the EEL probability map for a 100 keV electron beam (right) for a SMSERS-active trimer. Simulation of the plane-wave excitation is performed *via* the Discrete Dipole Approximation (DDA) at a wavelength of 532 nm. The wavevector of the excitation field is directed along the *z*-axis and is polarized along the *x*-axis. The 2D slice displayed corresponds to the plane where the electric-field magnitude is maximized. Other polarizations, wavevector directions, and projection planes were examined and show similar localization of the field in the junction regions. The loss-probability map, computed *via* a modified electron-driven DDA (e-DDA), is displayed at a corresponding loss-energy of 2.3 eV. In agreement with the experiment, the EEL map does not show an intense loss probability in the junction region. (c) Induced polarization maps (2.3 eV) obtained for two different positions of the electron beam (green bullet). Placement of the electron beam in the junction leads to a net antibonding arrangement of dipoles (right), whereas placement of the electron beam on the outside right corner leads to a net bonding arrangement (left). Also shown is the induced polarization (red vectors) and resulting scattered electric field (blue vectors), both normalized to unity to aid visualization. Both panels display 2D slices taken from fully 3D simulations of the trimer. The plane of visualization was chosen to lie at the height of the centroid of the two cubes. Reprinted with permission from Mirsaleh-Kohan (2012) © American Chemical Society.^140^ E. Low-energy electron microscopy (LEEM) and photoemission electron microscopy (PEEM) for plasmonic characterization of triangular Ag microstructures (a) Schematics of LEEM and multiphoton PEEM experiments. (b) Single photon PEEM image illuminated by UV lamp showing the Ag(111) crystal shape. The Greek letters label the island edges, where the dominant SPPs are coupled. In all experiments, the incident field *k*-vector is normal to the α edge. (c–h) Two-photon PEEM images showing beating patterns on the Ag crystal due to interference of the excitation light (*λ* = 460 nm) with SPPs. Dashed lines indicate the island geometry and regions of integration of the two-photon photoemission signal (c). The dashed arrow indicates the line along which the field interferences are considered (d). The excitation laser light is incident from the bottom at *θ* = 70° from the surface normal (green arrow) with linear (c–f) and circular (g and h) polarizations, which cause the asymmetric two-photon PEEM images in (e)–(h). The white arrows in (c)–(f) indicate projections of the linearly polarized incident light onto the surface plane. In (g) and (h) the white circulating arrows show the helicities of circularly polarized light; the red arrows, the corresponding in-plane directions of their spin angular momentums (SAMs); the white linear arrows, the *k*-vectors of SPPs; and the yellow arrows, their transverse SAMs. Reprinted with permission from Dai (2018) © American Chemical Society.^160^

Madsen (2017) *et al.* found that Cr and Ti adhesion layers, in a gold nanodisk architecture, dampened plasmonic excitations and thus reduced near-field electric field intensity and SERS signal (Fig. 4C). This was corroborated with EELS.^139^ This work also highlights a specific benefit of EELS to determine SERS: it allows the potential identification of plasmonic ‘dark’ excitations *i.e.* sub-radiant modes.^141–147^ which describe plasmon resonances that are not optically accessible, or only slightly so, but could be excited through near-field coupling,^148,149^ and thus influence SERS. Dark modes, having negligible net dipole moment, can be used to confine energy effectively in the near-field, suppressing re-radiation to the far-field.^141^ Elsewhere, Morla-Folch (2014) *et al.* examine gold nanostars with SERS and EELS, concluding that slight blue-shifts in LSPR spectral position for LSPs in the EELS spectra, at nanostar tips and ‘core’ region respectively, is as a result of a variation in the dielectric environment, meaning some care is needed in combining SERS and EELS results (Fig. 2C and 4B).^150^ While small resonance wavelength shifts are desirable in the technique of surface plasmon resonance biosensing, which uses a functionalized plasmonic surface, and monitors the reflected light, such resonance changes are not desirable in SERS where the resonant spectral position should be fixed. Further, we note that simulated EELS has also been used to understand better electronic spatial non-locality in plasmonics, crucial in the concentration of light on the sub-nanometre scale.^151^ Thus, EELS can be a valuable technique for characterizing all kinds of SERS media by showing the presence of dark modes in well-ordered analytical nanostructured substrates on top of providing the nanometric resolution to explore finer gaps and hotspots, even as a function of wavelength.^152^

Mirsaleh-Kohan (2012) *et al.* have interrogated nanoparticle dimers with EELS in the context of revealing inter-particle hotspots (Fig. 4D)^140^ that are believed to be responsible for single-molecule SERS (SM-SERS) phenomena.^153,154^ The SM-SERS capability of the system was verified experimentally *via* bi-analyte isotopologues.^155^ Many different numerical solver algorithms are now available to the SERS researcher, including the commonly used finite element method (FEM) or finite difference time domain (FDTD) approaches. Here, the authors use a discrete dipole approximation (DDA) model, which is less versatile than FEM or FDTD but suitable for modeling the behavior of multiple interacting dipoles in small, irregularly shaped nanoparticles. While a conventional DDA numerical model displays a high electric field in the gap, a modified ‘e-DDA’ numerical model, accounting for the electrons as the exciting vehicle, is also used, showing enhanced near-fields at the nanoparticles’ peripheries, which is in better agreement with the authors’ EELS experiment. Thus, the study raises doubt to the suitability of using experimental EELS to investigate plasmonic systems (of any kind). Plane wave excitation by light, which is uniform across the entire nanoparticle area, is not necessarily comparable to localized electronic excitation, which is highly anisotropic.^129^ A computational analysis of the difference between photonic and electronic excitation of plasmon modes has been explored in monomer and dimer metal nanorods by Bigelow (2012) *et al.* (Fig. 4A).^156^ Apart from the difficulty in correlating EELS and photon-induced plasmons, the high energy electron beam in EELS, here considered as an isolated technique, also may cause sample damage.^143^ Thus, it must be performed under vacuum, and like TEM, EELS is limited by sample thickness requirements.

### Photoemission electron microscopy

Photoemission electron microscopy (PEEM/PEM) is a surface characterization technique that uses electron emission from near-field hotspots after multi-photon absorption and secondary electrons are detected. Spatial resolutions as low as 10 nm can be achieved.^157^ El-Khoury (2014) *et al.* have evaluated the SERS performance of a nanometrically separated array of (∼10 nm diameter) silver nanospheres with PEEM and TEM, noting that the relative uniformity of near-fields results in reproducible SERS.^158^ Ji (2017) *et al.* uncover an optically inactive plasmon mode in a gold nanoring structure with PEEM, pointing to use in SERS.^159^ Awada (2016) *et al.* employ PEEM to illuminate the hotspot regions in a gold semi-continuous film, superimposing the PEEM image on top of a low-energy electron microscopy (LEEM) image in order to align regions of large electric field with the topography.^157^ Detailed plasmonic analyses are possible, for instance, Dai (2018) *et al*. have investigated the mechanics of interacting propagating plasmon modes, surface plasmon-polaritons (SPPs), in triangular silver microstructures with linear and circularly polarized light (Fig. 4E).^160^ PEEM for plasmonic imaging has recently been reviewed by Dąbrowski (2020) *et al.*^161^

Therefore, electron beam spectroscopies provide a useful avenue to investigate both analytical and HSD SERS platforms. Elsewhere, Edwards (2011) *et al.* reported cathodoluminescence, the reciprocal process to the photoelectric effect, to study LSPs in silver nanocubes, albeit without reference to SERS.^162^ The energy, spatial and temporal resolutions of scanning near-field optical microscopy (SNOM), EELS, PEEM and cathodoluminescence techniques have been summarized recently by García de Abajo and Di Giulio (2021) in a broad perspective on the utility of electronic excitation in the analyses of photonic structures.^152^ Moreover, a report has surfaced lately combining EELS with a scanning tunneling microscopy set-up but biased for field emission rather than in the tunneling regime.^163^ The authors demonstrate dual-mode spectroscopic and topographical imaging of μm-scale Au and Ag Island formations, as well as nm-scale roughness features, indicating suitability for analysis of different regimes of features on plasmonic substrates. However, note is made that high tip–surface biases induce roughening, which could compromise analysis of nanometric features (here 20 V, 600 pA@2–5 nm on Au(111)). Whether used as part of a multi-modal system or separately, scanning probe microscopies confer another opportunity for SERS substrate analysis.

## 4. Scanning probe microscopy

### Near-field scanning optical microscopy

**Fig. 5:**
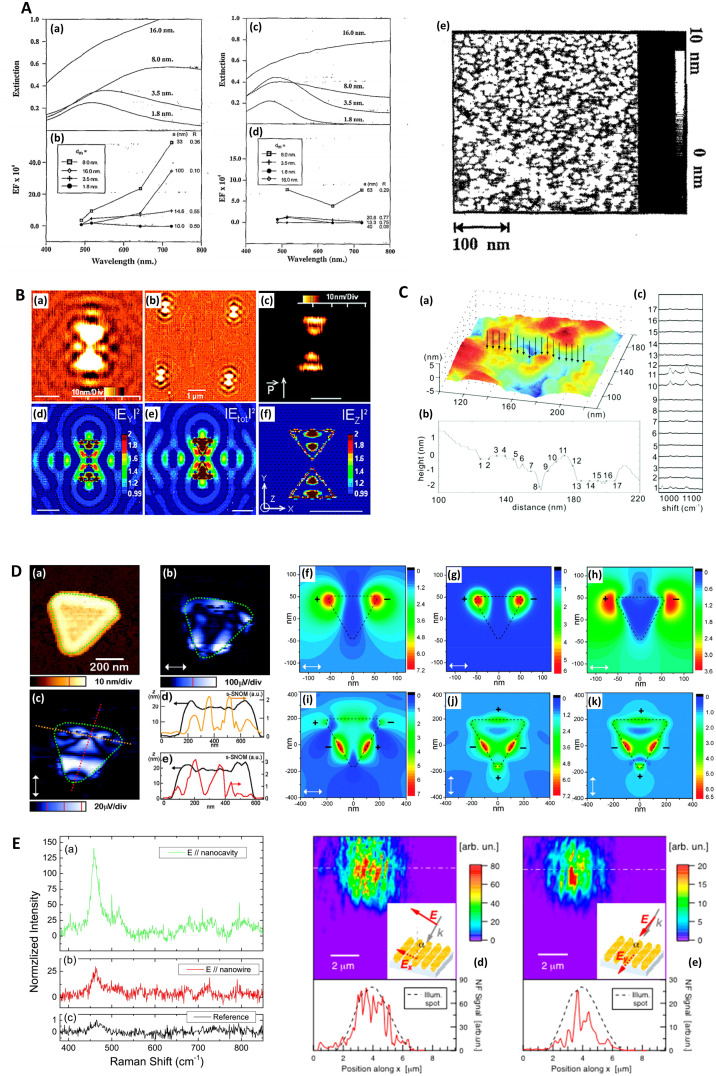
Probe microscopy in SERS substrate characterization. A. Silver island films for SERS analyzed with atomic force microscopy (AFM) (a) Unpolarized, normal incidence extinction spectra of “as-deposited” Ag island films with thicknesses at 1.8, 3.5, 8.0, and 16.0 nm; deposition rate is 0.3 nm s^−1^. (b) SERS EFs for the same films at laser excitation wavelengths 488.0, 5 14.5, 641.3, and 722.0 nm. The values of thickness, *d*_m_, major particle diameter, *a*, and minor-to-major axis ratio, *R*, are listed from top to bottom in order of decreasing EF at *λ*_ex_ = 722 nm. (c and d) Same films and conditions as in (a and b) but films annealed at 600 K for 60 minutes. (e) AFM image of as-deposited Ag island film on glass slide; prepared at room temperature. Deposition rate is 0.3 nm s^−1^, thickness is 3.5 nm. Reprinted with permission from Van Duyne (1993) © American Institute of Physics.^174^ B. Molecular-motion-induced photochemical imaging for imaging of plasmonic near-fields. (a)–(c) AFM images of the sample surface after irradiation. (c) Obtained by adjusting the contrast level on image (a). The incident polarization, represented in (c), was parallel to the bowtie major *Y*-axis. Intensity |*E*|^2^ calculated contour plots for *Y*-polarized incident light in a plane 5 nm above the particle surface: (d) |*E*_*Y*_|^2^, (e) total intensity, (f) |*E*_*Z*_|^2^. The color scale in (d)–(f) is such that brown is high and blue is low. The scale bar represents 500 nm unless otherwise noted. Reprinted with permission from Hubert (2008) © American Chemical Society.^175^ C. Nanoscale roughness on metal surfaces can increase tip-enhanced Raman scattering by an order of magnitude. (a) Tip-enhanced Raman spectroscopy (TERS) mapping on a rough Au surface. A scanning tunneling microscopy (STM) image of the sample is shown in (a). TERS data was collected at the positions indicated by the arrows. The cross-section of the topography image is shown in (b), and the TERS collection sites are labeled with crosses. Panel (c) is the corresponding TERS sequence. The numbers denote the sites where the spectra were collected. The exposure time of each spectrum was 30 s, and the laser power was 0.5 mW. Reprinted with permission from Zhang (2007) © American Chemical Society.^195^ D. Near-field scanning optical microscopy (SNOM) to illuminate higher-order plasmonic modes in silver nanoprisms. (a) Topography and corresponding tip-scattered near-field images at 633 nm for (c) p- and (b) s-polarization, of a large single crystal Ag nanoprism exhibiting quadrupole excitation. The scattering SNOM cross-sections (d and e) indicate spatial field variations at length scales as short as 20 nm. (f)–(k) Calculated optical near-field distribution of the Ag nanoprism for 633 nm excitation. Top row (f–h): dipolar mode for nanoprism with edge length 120 nm, thickness 35 nm, and 10 nm truncated from each tip under s-polarized excitation, with total field |*E*^2^| = |*E*_*x*_^2^ + *E*_*y*_^2^ + *E*_*z*_^2^| (a), *z*-component |*E*_*z*_^2^| (b), and in-plane field |*E*^2^| = |*E*_*x*_^2^ + *E*_*y*_^2^| (c). Bottom row (i–k): quadrupolar fields of nanoprism with edge length 450 nm, thickness 25 nm, and 35 nm truncated from each tip. Panel (d) shows the total field |*E*^2^| under the s-polarized excitation. Panels (j) and (k) represent the total field |*E*^2^| and *z*-component |*E*_*z*_^2^| under p-polarized illumination, respectively. Signs represent the relative phase of quadrupoles. Reprinted with permission from Rang (2008) © American Chemical Society.^168^ E. Gold nanowires (NWs) analyzed via SERS and near-field optics. (a) NIR (785 nm) SERS and Raman spectra of methylene blue deposited on the NWs sample (a and b) and (c) on a reference gold sample. (a) SERS signal with excitation field polarized along the nanocavities axis (*θ* = 0). (b) SERS signal with excitation polarized along the NWs long axis (*θ* = *π*/2). (c) Raman signal acquired on the reference. Experimental conditions are the following: (a and b) *P* = 43 μW, integration time *T* = 240 s; (c) *P* = 4.3 mW, *T* = 600 s. The signal intensities in panels (a) and (b) are normalized to the reference, taking into account the different powers and integration times. The peak intensity at 445 cm^−1^ of the reference signal (c) is normalized to one. The reference signal is *ca*. 2 orders of magnitude smaller than the one measured at 633 nm. (d and e) Near-field optical maps acquired on the same region of the sample illuminated with two mutually orthogonal polarization directions (see insets). Line profile analysis along the dashed-dotted segments is plotted at the bottom of the maps. The intensity is normalized to the signal averaged over the whole maps. The displayed features represent the details of the scattered spot, collected in the near field. Reprinted with permission from D'Andrea (2014) © American Chemical Society.^169^

Near-field scanning optical microscopy (NSOM/SNOM) uses a nanoscale tip that is illuminated with laser light and placed proximal to a surface to break the diffraction limit for high-resolution imaging, on the 10 nms-scale. It may be performed in apertured *i.e.* a waveguide with aperture size ≪*λ*, or in an apertureless/scattering mode^164^ with an atomic force microscopy (AFM) tip; indeed, SNOM benefited from the development of AFM in the 1990s.^165^ Various authors have used SNOM imaging to characterize plasmonic near-fields, as pointed out in ref. 166. Esteban (2011) *et al.* used apertureless SNOM to study quadrupolar plasmonic resonances in nanodisks,^167^ while Rang (2008) *et al.* similarly investigated the near fields of Ag nanoprisms and found that sharp geometrical features, triangle tips in this case, do not necessarily represent the locale of highest enhancement whenever multipole resonances are involved (Fig. 5D).^168^ The authors note the importance of understanding the precise characteristics of near-field profiles in nano-antennae for SERS purposes. D'Andrea (2014) *et al.* correlated SERS and SNOM data in a study of EM-interacting gold nanowires finding optimal enhancement 2× at 785 nm than 633 nm, and nearly three orders of magnitude greater for longitudinally polarized light *versus* transverse illumination (Fig. 5E).^169^ More recently, Kusch (2017) *et al.* have employed an AFM tip in a scattering SNOM setup to monitor near-fields in plasmonic hotspots at both the laser excitation wavelength and Raman de-excitation wavelengths.^166^ The importance of consideration of the EM profile at the Raman-shifted de-excitation wavelength would often appear to be lost in many modern SERS substrate characterization studies, perhaps compromised by the allure of the simplicity of the common *E*^4^ approximation, as has been discussed in ref. 170 by Schatz, Lombardi, Dawson and Deckert.

In a notable study, Bouillard (2010) acquired spectra at regular intervals in a periodic nanodome array to record a hyperspectral plasmonic image.^231^ This then permits a full experimental characterization of the plasmonic response of the system at every point, without the interference of analyte molecules being used as vehicles to understand the near-field, and is perhaps limited only in utility to SERS studies by the intricacy of the experimental set-up.

### Atomic force microscopy

Atomic force microscopy (AFM) differs from other scanning probe microscopies in that it is a non-optical technique, relying on the deflection of a cantilevered tip at the sample surface to assess surface profiles. AFM may be operated in several modes: contact, non-contact and tapping, which depend on the resolution required and the specific material. The technique is suitable for an accurate 3D topographical assessment on the 10 nm-scale up features on lithographically produced SERS nanostructures although it is not appropriate whenever individual structures are too proximal and have a high aspect ratio,^55^ or are too complex.^40^ Moreover, it is normally slower than scanning tunneling microscopy (STM) as STM operates in constant current mode. AFM is not limited by optical diffraction and can achieve sub-nanometer resolution, and thus further allows an estimation of the surface roughness, or more grainy features, the precise morphology of which may be crucial with regard to the magnitude of any observed SERS.^113,171–173^ AFM therefore may also be considered a good choice for the characterization of HSD SERS substrates, although for accurate nanometric feature measurements the feedback control parameters must be precisely tuned.

AFM was first used to interrogate the morphology of SERS substrates by Van Duyne (1993) *et al.*, where the authors explored annealed silver island films (Fig. 5A). Interestingly, in a precursor to a future focus on more rationally designed SERS media, the authors noted that the 10^7^ EF, comparable to planar rough SERS substrates, may have a significant component (10^2^) arising from propagating surface plasmon-polariton (SPP) generation, not just localized modes.^174^ AFM has also been used to map EM near-fields in 3D around nanostructures *via* use of a light-sensitive polymer, specifically, with silver bowtie structures by Hubert (2008) *et al.* (Fig. 5B). Movement of a probe molecule, embedded in the polymer medium, as a result of local near fields, alters the polymer topography.^175^ This technique potentially allows a comprehensive understanding of the plasmonic activity across a nanostructured area, and thus a full indication of a SERS sensor's analytical sensitivity and signal uniformity. Other interferometry and stylus-based profilometry approaches exist, but typically have much poorer lateral resolutions (10–10^2^ × poorer) and may have material restrictions. Nevertheless, these may be useful for verifying plasmonic layer thickness, typically 10 s–100 s nm.

### Scanning tunnelling microscopy

Scanning tunneling microscopy (STM) uses a near-surface tip, relying on quantum tunneling between tip and a conductive surface for atomic resolution of surface roughness or local electron structure. Roughened electrodes were the first SERS platform studied, albeit with the increase in the Raman enhancement initially misattributed as being primarily due to surface area increase of the electrode. Aloisi (1994) *et al.* studied the roughness in silver (111) films with STM,^176^ after oxidation–reduction cycles, noting potentials of −0.5 V and lower produced optimal SERS response with surface features on the order of 10 nm, in line with observations elsewhere.^113^ Ti_2_N can potentially provide plasmonic enhancements comparable to gold but at lower cost. Soundiraraju (2017) *et al.* use both STM and AFM in the characterization of few-layered Ti_2_N substrates for SERS purposes, noticing the hexagonal close packing of titanium with STM, which relates to plasmon quality, and measuring the 3D profile of a Ti_2_N flake with AFM. The authors further use AFM to assess the surface roughness of a paper substrate, used as the SERS base layer, pre- and post Ti_2_N application.^125^ In a fundamental study, Dawson (1991) employed STM in SERS to probe the relationship between surface roughness on silver surfaces and observed SERS enhancements, concluding that, in fact, a decrease in SERS signal was observed not as a result of surface roughness changes, but of increased grain boundary density, which act as perturbative centers for SPPs.^172^ In addition to the usefulness of scanning probe microscopies to characterize metal film surfaces, their use in SERS studies is also associated with using the tip as an active part of the system to form and control hotspots: tip-enhanced Raman spectroscopy.

### Tip-enhanced Raman spectroscopy

Tip-enhanced Raman spectroscopy (TERS) employs a scanning probe set-up and a plasmonic tip, from a scanning probe microscopy set-up, and circumvents two problems with conventional SERS, namely, 1. fewer molecules can be interrogated, and 2. it is not diffraction-limited *i.e.*, better resolution,^177–182^ as low as 4 nm.^183^ The technique, viewed as the ‘spectroscopic cousin’ of AFM and STM, arose as a type of apertureless near-field optical microscopy, differing where instead of using a sub-diffraction aperture, a plasmonically active scattering tip is illuminated with obliquely oriented light.^184^ The limitation of TERS is in control of tip fabrication,^185,186^ and, in fact, has always been considered the deciding factor in the success or failure in near-field optical experiments.^184^ In many ways, TERS is not just an offshoot of SERS research but now a research area of its own.^185,187–190^

Besides the clear application to single molecule analysis—indeed this would appear to be the angle of most TERS studies and actually, Stöckle (2000) *et al.* who were the first to report TERS did so with an analysis on bucky balls^182^—TERS can also be used for SERS substrate characterization. Therefore, the properties of TERS systems must be properly understood, and to this end, Yang (2009) *et al.* modeled the 3D distribution of EM profiles in TERS, varying tip–sample separation, tip size, sample material, and the polarization and incident angle of the impinging plane wave. The authors note the pre-eminent importance of tip–sample distance on TERS sensitivity and tip sharpness on spatial resolution, as well as an optimal incident illumination angle of 40–60°.^191^ The authors explain that a vertically oriented field – with wavevector direction perpendicular to the substrate interfacial plane *i.e.*, pointing directly downwards – upon reflection, provides opportunity for increased destructive interference with the incident EM field. Greater emphasis on the impact of tip material and morphology on the plasmonic enhancement and spatial resolution is necessary.^192^ Dawson (2017) *et al.* presents a model description of plasmonic modes in a TERS set-up, explaining their location in the visible and NIR spectral ranges as a result of a non-linear modification to the local dielectric properties of gold by the strong TERS-induced dc-field.^193^ The broad applicability of such studies is that, in order to properly understand the enhancement in coupled plasmonic media, the precise experimental configurations and nano-feature morphologies must be considered. Recently, Cheng (2021) *et al.* have communicated a theoretical treatment of resonance TERS (‘TERRS’) and plasmon–exciton (*i.e.* plexciton) coupling to extend the enhancement of Raman dye molecules to both Stokes and anti-Stokes Raman-shifted photons,^194^ which is not possible with (pure) plasmonic enhancements.

TERS relies on a single hotspot: the gap between the sample surface and the tip and thus, rough surface features can be isolated and their plasmonic nature analyzed. This means TERS is appropriate for analysis of not only the near-fields associated with nanostructures on the 10 s nm-scale, but also finer features aligned with HSD substrates. Notably, Zhang (2007) *et al.* showed enhancement changes by a factor of 10× in a TERS-STM system when the surface morphological features of size 1–2 nm were interrogated (Fig. 5C),^195^ and Awada (2016) *et al.* have introduced functionalized tip–surface enhanced Raman scattering (FTERS) as a way to measure near-field hotspots *via* attached Raman-active molecules to a TERS tip. Although a gold tip is used, the authors note that a plasmonically inactive material may better evaluate surface hotspot measurements.^157^

## 5. Wavelength-scanned surface-enhanced Raman excitation spectroscopy

**Fig. 6:**
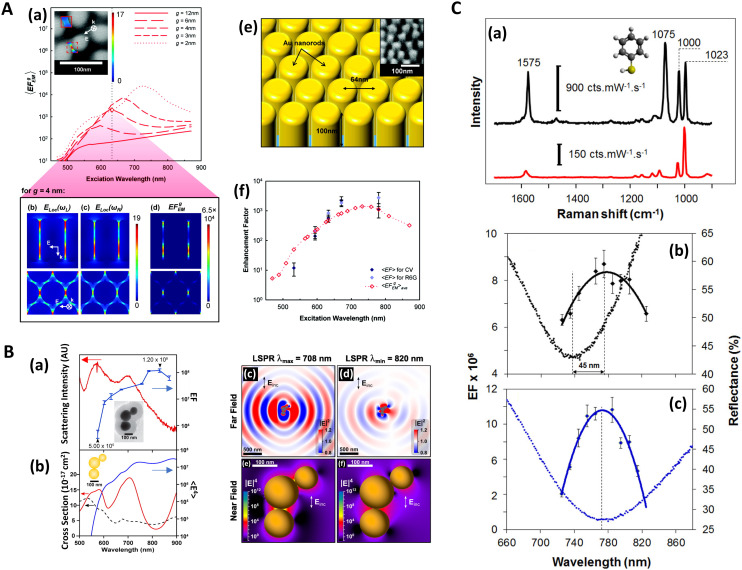
Multiwavelength SERS characterization studies. A. Multiwavelength SERS study of gold nanorods. (a) Surface-enhanced Raman excitation spectroscopy (SERES) profiles, 〈EF^g^_EM_〉, for nanorods with diameters, *d*, between 52 and 62 nm (inter-rod gaps, *g*, 12 to 2 nm, respectively) calculated for a Stokes Raman shift of 850 cm^−1^. Inset shows two examples of calculated local electric field distributions between two nanorods superimposed on an SEM image of the substrate (solid box, *g* = 12 nm; dashed box, *g* = 3 nm). 〈EF^g^_EM_〉 spectra are plotted with points at ∼20 nm intervals, with additional points at the laser excitation wavelengths. (b–d) Cross-sections of the nanorod array showing calculated distributions of electric field at excitation and scattering frequencies and Raman EF in the gap regions between rods for the case *d* = 60 nm, *g* = 4 nm. The cross-sections with the short axis of the rods in-plane (lower images) are taken at the peak field regions near the top of the rods. *E* and *k* indicate the polarization and wavevector of the incident radiation. All *E*-field and EF distributions shown are for an excitation wavelength of 633 nm (indicated by dotted vertical line in (a)), and incident *E*-field magnitude of unity. (e) Schematic of the model Au nanorod array after removal of a porous alumina template. Inset shows an SEM image of the surface taken at 40° incidence. (f) Graph comparing calculated average EF, 〈EF^g^_EM_〉_ave_, for a Raman shift of 850 cm^−1^ and the observed EF 〈EF〉 of CV and R6G Raman dyes at 915 and 775 cm^−1^, respectively. The calculated EF assumes a uniform distribution of gap widths in the range 1.5–20.0 nm. Reprinted with permission from Doherty (2010) © American Chemical Society.^42^ B. Experimental and calculated correlated HRTEM–LSPR–SERES data for the nanoantenna trimer. (a) Experimental dark-field scattering spectrum (red) and excitation profile for the 1200 cm^−1^ band (blue) of the trimer. Inset: micrograph of trimer structure. (b) Electromagnetic modeling of the nanoantenna trimer, displaying the 〈*E*^4^〉 enhancement in solid blue, scattering in solid red, and absorbance in black dashed line. Both experimentally and theoretically, the trimer provides maximal SERS enhancement, where the far-field scattering is relatively weak. (c and d) Far- (top row) and (e and f) near-field (bottom row) electromagnetic interactions for peaks and troughs in the LSPR spectrum for the nanoantenna displayed (a and b): (c and e) *λ*_ex_ = 708 nm and (b and d) *λ*_ex_ = 820 nm. The lower panels show the large electric field enhancement in the gap region between touching spheres. For both wavelengths the near-field intensity is very similar, manifested in similar EFs. The far-field scattering intensity is smaller at 820 nm than at 708 nm, manifested in different LSPR intensities at the studied wavelengths and illustrated by a difference in the light waves emanating from the particle upon irradiation. Reprinted with permission from Kleinman (2013) © American Chemical Society.^92^ C. Multiwavelength SERS study of film over nanosphere (FON) structures. (a) SERS (black) and normal Raman (red) spectra of benzenethiol acquired with 785 nm excitation, power = 50 μW, and time = 10 s. (b and c) Near-field profiles (EF, solid line) and far-field optical responses (reflectance, dotted line) of (b) stationary film over nanosphere structures (ST-FONs) and (c) spun film over nanosphere structures (SP-FONs). Reprinted with permission from Kurouski (2017) © American Chemical Society.^89^

Most SERS studies employ one or two excitation wavelengths, often motivated by the resonance of a specific analyte, fluorescence mitigation, or simply limited laser availability. However, multi-wavelength experimental SERS studies can prove profitable, seeing the discrepancy between the near-field (of relevance to SERS) and far-field (white light optics) and difficulty in the computational simulation of complex nanostructures (Fig. 6). Wavelength-scanned surface-enhanced Raman excitation spectroscopy (WS-SERES or SERES) has been promoted by the late Richard Van Duyne, the importance of which he stressed at a 2017 Faraday Discussion in SERS^196^ and in the associated Spiers Memorial Lecture.^197^ Despite this, SERES is an underused technique, perhaps due to the onerous nature of taking SERS measurements with multiple lasers, or indeed the simple availability of many excitation wavelengths, whether individual light sources or *via* a suitable tunable laser.

Gregory (2001) *et al.*, in the context of electronic image potential states in alkanethiol films, may have been the first to perform a focused SERES study,^198^ as indicated in ref. 199, although SERS excitation spectra are also discussed by Otto (1992) *et al.* in an early SERS review article,^200^ and Van Duyne (1993) *et al.* used multiple laser wavelengths in a study into the roughness effect on SERS in silver films.^174^ In a well-known 2005 publication, Van Duyne and co-workers used SERES to enforce the EM mechanism of SERS as the dominant enhancement pathway. The study emphasized the importance of considering the (analyte/vibrational bond specific) Raman-shifted wavelength in SERS enhancement and its overlap with the nanostructure LSPR(s) spectral positions.^201^

More recently, the same authors have investigated roughened silver and gold film-over-nanosphere (FON) SERS structures^89^ with multiple laser wavelengths, and Doherty (2013) *et al.* have used SERES to probe near-field far-field relationships in SERS for proximal nanopillars (Fig. 6A and C).^90^ Khanafer (2016) *et al.* have shown the SERS response in silver nanoparticle rings in micro/nano-porous polymethyl methacrylate (PMMA) *via* excitation at 457, 488, 514 and 633 nm, finding an EF of 3.8 × 10^8^ at 488 nm for the optimal ring dimensions *via* Raman dye *trans*-bis-(4,4′-bipyridyl) ethylene (BPE).^202^ SERS platforms with highly EM-coupled nanostructures with dimensions on the visible-range scale, and thus typically red-shifting resonances as system size increases, may benefit from excitation in the near-infrared range (785 nm, 830 nm *etc*.),^197^ which could be explored with SERES. Therefore, despite an infrequent appearance in SERS substrate characterization, SERES would appear to be a valuable approach for the full characterization of novel SERS substrates, whether they be analytical or of more HSD character. Indeed, significant enhancements beyond that expected from far-field optics or pristine *i.e.* without the inclusion of roughness, numerical simulations, may indicate more HSD-like behavior where hitherto undetected hotspots are dominating the SERS response across a wide wavelength range.

SERES studies on substrates that are clearly dominated by hotspots arising from imperfect fabrication conditions may be useful. Here, a range of erratically shaped features and/or random surface asperity shapes support plasmon-polaritons at many wavelengths. In these cases, SERES can be used to find out the spectral profile of the SERS enhancement – as opposed to the enhancement at ‘one’ resonance wavelength. In fact, this may be where SERES is most useful. Finding the enhancement as a function of wavelength due to a distribution of nanometric features is difficult to understand with simulation. Aside from gap plasmons confined to small surface features, the main consideration for optimum SERS might simply be the material quality factor, calculated from the dielectric function for the chosen plasmonic metal, and at a given wavelength. Alternatively, sharp features with erratic morphologies, typically associated with bottom-up fabrication processes, can be the primary contributor to the SERS enhancement, but here, the concentration of electric fields is purely geometrical *i.e.*, the non-resonant lightning rod effect, and thus of less importance to a wavelength-scanned study. Sharp surface features may however be critical elsewhere, such as in affecting the substrate–solution interaction.

## 6. Substrate hydrophobicity

Another overlooked area of SERS substrate characterization is the response of the nanostructured platform to the analyte solution. The nanostructured area is not flat and thus can result in hydrophobic interactions. We note that the areal increase may be considered by calculating the average surface area of a typical nanostructure and the nanostructure surface density, but that this does not capture the precise nature of any potential hydrophobic response. In a historical work, Young (1805) derived an expression for the contact angle, *θ*_C_, the angle between substrate interface and microdroplet curvature away from this surface, in terms of the three interfacial energies. The modern seminal hydrophobicity papers date back to the 30 s and 40 s and received little initial attention before being rediscovered decades later.^203^ Wenzel (1936) considered surface roughness as impacting contact angle,^204^ and later, Cassie and Baxter (1944) considered composite surfaces, normally thought of in terms of solid material and air gaps.^205^ Since then, the validity of these simple models has been debated^206,207^ and a greater understanding of the complexity of the wetting in nanostructured–microstructured surfaces has emerged^208^ Contact angles can be measured with an optical goniometer where an applied droplet is imaged *via* a high-resolution camera. Where necessary, then, surface tension can be calculated (tensiometry).5
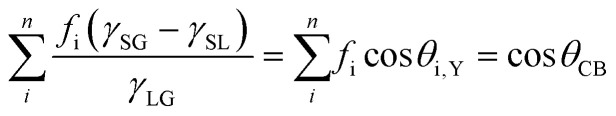
Cassie and Baxter's expression relating the surface energies at the solid–gas (*γ*_SG_), solid–liquid (*γ*_SL_), and liquid–gas (*γ*_LG_) for *n* material fractions (*f*_i_) to the Cassie–Baxter contact angle, *θ*_CB_.

As SERS entails surface texturing, then evaluation of the response of the plasmonic surface to applied analyte is important. Drop-casting, that is applying a microlitre-sized droplet to the surface *via* micropipette, is still the dominant method of analyte application, being quick and easy albeit sacrificing some control over molecular surface deposition,^110,209^ which can have a profound effect on subsequent SERS measurement sensitivity.^210–212^ The permeability of the analyte-containing aqueous phase can impact whether target molecules are close to LSP modes or in the case of a HSD SERS substrate, near the rough or sharp features. Similarly, suboptimal wetting can increase the likelihood of multilayer analyte formation. We note that the contact angle response of a nanostructured surface is not only dependent on the substrate but is a convolution of this and the solution applied, which itself is contingent on the presence of (and concentration of) the target molecules (the solute). Therefore, solutions used in initial hydrophobicity substrate characterization should match those used in the actual SERS experiments. The final distribution of particulate matter in a drying droplet is also dependent on other factors not relating to the SERS-active nanostructured surface, for instance, the ambient medium, which can cause hydrothermal waves in drying drops.^213^ The complex behavior of drying drops for biomedical application was highlighted by Sefiane (2010), but with a focus on the large-scale patterns in pathological serum samples (‘Litos tests’),^214^ while recently, we have reviewed the process in the context of biomarker SERS analysis of saliva samples, noting that while analyte inhomogeneity in drop-casting may be overlooked, simple air-dried samples are nonetheless useful for advancements in portable point-of-care diagnostics.^22^

The measurement of substrate–analyte interaction is no more evident than in studies where the SERS substrate is designed specifically for superhydrophobic (*θ*_C_ > 150°) purposes, usually with the aim to concentrate the analyte molecules and increase sensitivity. Song (2014) *et al.* fabricated a radial-strip bulls-eye structure that induces a superhydrophobic response. Here, the authors apply 60 nm gold colloids, which densely aggregates in the 100 μm central bullseye region, before depositing a second droplet with the analyte solution, detecting Rhodamine 6G (R6G) to femtomolar (10^−15^ M) concentrations. While interparticle spacing is not controlled, the authors envisage using a silica shell in future experiments *i.e.* shell-isolated nanoparticle-enhanced Raman spectroscopy (SHINERS).^215^ De Angelis (2011) *et al.* explored textured nanocylinder and nanocone arrays detecting R6G down to the attomolar (10^−18^ M) level.^216^ Yang (2016) *et al.* took a different approach to analyte enrichment by mitigating contact line pinning of drying droplets to concentrate analyte molecules, achieving quantitative detection of R6G down to ∼75 fM. This is performed by slippery liquid-infused porous SERS (SLIPSERS) substrates consisting of perfluorinated fluid sprayed onto Teflon membrane-on-glass, or siliconized bowl arrays. The authors observe the phenomenon *via* a goniometer with microscale particles and polystyrene beads in ethanol, but the effect also applies to sub-nanometric molecules and nanometre-sized colloids.^217^ We note, from this procedure is that additional layers can shift the plasmon peak position, which depends acutely on adjacent dielectric conditions – the premise of surface plasmon resonance sensing – and if possible, should be numerically modeled prior to treatment.

**Fig. 7:**
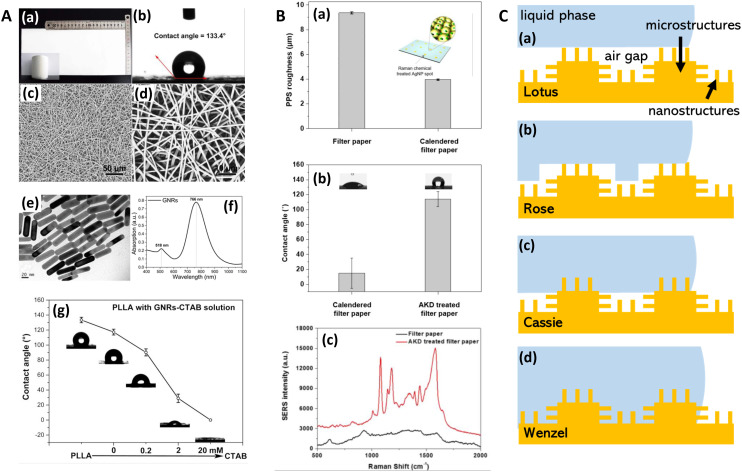
Hydrophobic substrate characterization in SERS. A. Hydrophobic fibrous paper-based plasmonic substrate for SERS. (a) Photograph of paper substrate: as large as 13 × 30 cm^2^ with high smoothness and uniformity. Inset: paper can be freestanding and flexible. (b) Contact angle measurement, and (c) low-magnification and (d) high-magnification SEM images of PLLA nanofibrous paper prepared *via* electrospinning. (e) TEM image and (f) absorption spectra of gold nanorods. (g) Contact angle measurements of PLLA nanofibrous paper after dropping GNRs–CTAB solution with increased CTAB concentration. CTAB = cetyl-trimethylammonium bromide. PLLA = poly(l-lactic acid). Reprinted with permission from Shao (2015) © American Chemical Society.^218^ B. Chemically treated hydrophobic filter paper for SERS sensing in pesticide detection. (a) Surface roughness analysis of filter paper and calendered filter paper (Parker Print Surf instrument). (b) Contact angle of calendered filter paper and alkyl ketene dimer (AKD)-treated filter paper. Inset: photographs are water droplets on calendered filter paper and AKD-treated filter paper, respectively. (c) SERS spectra of AgNP spots on filter paper and AKD-treated filter paper treated by 5 μL of1 μM 4-aminothiophenol solution. Black line: SERS spectra of AgNP spots on filter paper. Red line: SERS spectra of AgNP spots on AKD-treated filter paper. Reprinted with permission from Lee (2018) © American Chemical Society.^219^ C. Wetting behavior in different regimes on a hierarchical nanostructured surface (nm–μm range). (i) lotus, (ii) rose, (iii) Cassie and (iv) Wenzel. Further details of other regimes are given in Bhushan (2010).^208^ © M. Hardy 2021.

More economical strategies to achieve hydrophobicity are also apparent. Shao (2015) *et al.* used fibrous paper substrates loaded with gold nanorods to achieve wetting behavior approaching superhydrophobicity (133° *θ*_C_) (Fig. 7A). The authors detect R6G to 0.1 nM level and report 8% relative standard deviation (RSD).^218^ Lee (2018) *et al.* also employ a paper-based substrate but chemically treated with alkyl ketene dimer to induce hydrophobic wetting (114° *θ*_C_) with AgNPs (Fig. 7B). In doing so, a less pervasive regime is promoted, with AgNPs remaining near the surface rather than lying deeper within the fibrous paper structure. Pesticides Thiram (dimethylcarbamothioic dithioperoxyanhydride) and Ferbam (tris(dimethyldithiocarbamato)iron) were detected at LoDs of 0.46 nM and 0.49 nM respectively. An RSD of 6.19% was recorded (4-aminothiophenol).^219^

We observe that considerations of wetting behavior are not always necessary, for instance, in cases where swellable nanoparticle impregnated polymeric films are used,^52,220^ or more commonly, various kinds of microfluidic SERS systems with continuous fluid flow.^221–223^ Alternatively, solid analytes can also be depressed into a SERS-active medium for detection.^224^ Otherwise, the evaluation of substrate wetting behavior is pertinent for all kinds of nanostructured surfaces but may be especially important for hierarchical arrangements *i.e.* where features span the whole range of nano- and micrometer scales (Fig. 7C),^208^ and where it is not clear where analyte molecules preferentially deposit.

## 7. Discussion and future prospects

Although the presentation herein is wide-ranging in nature, we observe that many SERS characterization studies, and arguably the best ones, combine the relative advantages of various approaches to fully elucidate the nature of SERS media. Soundiraraju (2017) *et al.* performed a thorough characterization of a Ti_2_N-based SERS substrate, incorporating, high-resolution TEM, PEEM, SEM(–EDX), AFM, STM, and X-ray diffraction (XRD).^125^ Kleinman (2013) *et al.* have performed SERES, in a study into the near-field, far-field relationship between spherical gold dimers and trimers, supported by TEM analysis.^92^ Beshr (2021) gain spectroscopic and topographical information from STM-EELS,^163^ similar observations could be made with light-emission STM,^225^ which could offer improvements in energy resolution over EELS and spatial resolution improvement over cathodoluminescence measurements.^152^

**Fig. 8:**
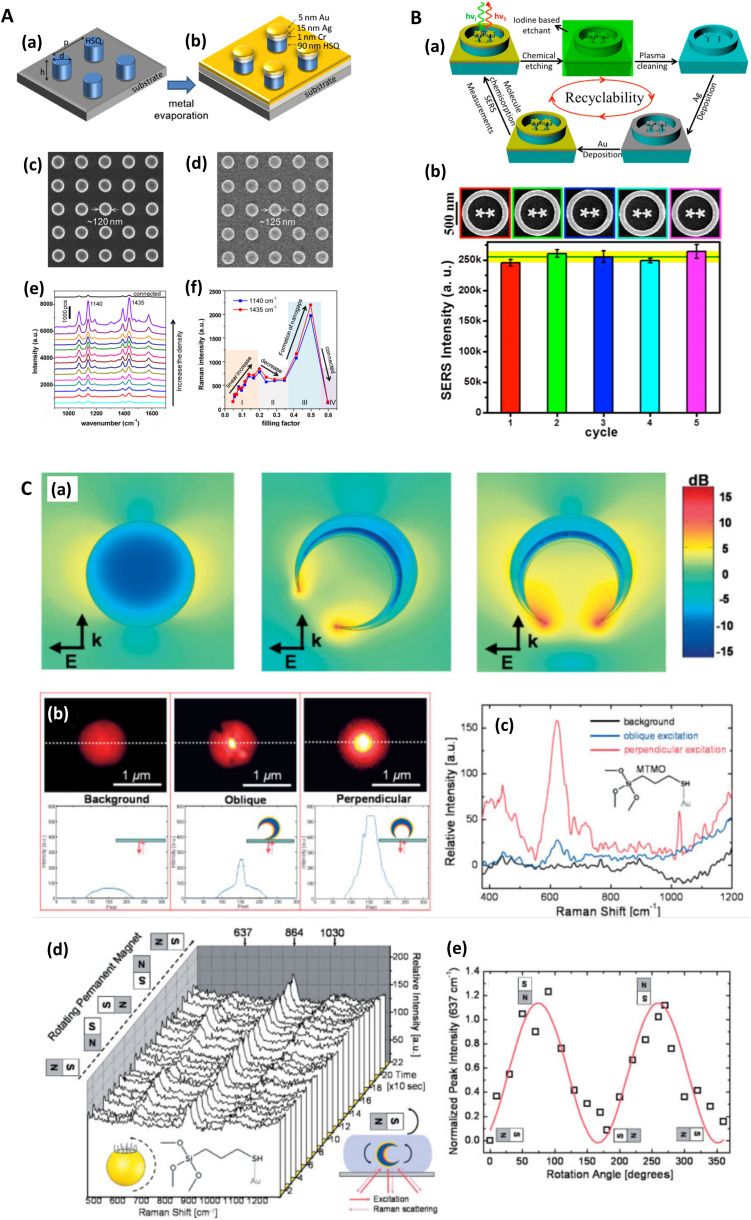
Fill factor, reusability, and orientation. A. The effect of nanostructure density/fill factor on SERS (a) Schematics of electron beam lithography (EBL)-defined hydrogen silsesquioxane (HSQ) nanoposts with diameter *d* and pitch *p*. (b) Schematics of the plasmonic nanostructures after depositing metal on HSQ nanoposts. (c) SEM image of HSQ nanoposts with a diameter of 120 nm and pitch of 240 nm. (d) SEM image of the plasmonic nanostructure array after metal deposition on HSQ nanoposts in (c). (e) Raman scattering spectra of plasmonic nanostructures with varied density. (f) The SERS intensity as a function of the particle density. Reprinted with permission from Wei (2016) © The Optical Society.^104^ B. Recyclable, ring-cavity-enclosed, bimetallic nanostars for SERS. (a) Illustration of the maskless recycling process that can be applied to 3D geometries such as AgAu-3D-nanostar-dimer-in-ring nanostructures. (b) SERS intensity of the *p*-aminothiophenol (*p*ATP) band at 1077 cm^−1^ recorded after each recycling process from the same sample. The pATP molecules were chemisorbed from 10 μM solution concentration, and the experimental parameters were 830 nm excitation source at 1.4 mW power and 30 s acquisition time. The corresponding SEM images for each recycling step are shown in the top panel. The green error bars show the standard deviation obtained from sets of 10 measurements recorded from different structures within the same array. Reprinted with permission from Gopalakrishnan (2014) © American Chemical Society.^227^ C. Magnetically modulated SERS detection of 3-mercaptopropyltrimethoxysilane (MTMO) molecules tethered on a single nanocrescent. (a) Simulated local electric-field-amplitude enhancement in dB by a single nanocrescent oriented 0° (right) and 45° (middle) with respect to the 785 nm light-incident direction, in comparison with an 80 nm Au nanosphere (left). (b) Intensity images and the cross-line intensity plots of a laser focal spot without a nanocrescent (left), and with a single nanocrescent obliquely (middle) and perpendicularly (right) oriented with respect to the direction of excitation laser light. (c) SERS spectra of MTMO molecules on the surface of the glass slide (background), and on the single nanocrescents with oblique and perpendicular orientations. (d) Series of SERS spectra as a function of time while continuously changing the external magnetic field direction. (e) Intensity plot of the 637 cm^−1^ Raman peak *versus* approximate rotational angles of the permanent magnet. Reprinted with permission from Liu (2005) © John Wiley and Sons.^63^

There are other aspects of SERS substrate characterization that we have left undiscussed but that are relatively simple to perform, and do not require any novel experimental apparatus. This includes the common measurements on substrate uniformity *i.e.* spot-to-spot on the same substrate, and reproducibility *i.e.* batch-to-batch measurement across different substrates,^34^ as well as SERS substrate reusability assessments, where applicable (Fig. 8B).^222,226,227^ The question as to what kinds of SERS media might be appropriate for analytical measurements is a much broader inquiry that still stimulates debate,^17,28,34^ but we would perhaps like to reiterate the sentiment of Bell, in Aitchison (2017) *et al.*, in that it depends on the requirements of the end-user, and that the requirement for calibration is no different to other analytical techniques.^16^ Similarly, this review does not address the problem of meaningful *cross-comparison* of SERS substrates, although arguably this is implicit in certain discussions, for example, discrepancies in near- and far-field optics. This is an ongoing problem in SERS, evidenced by the aforementioned, large-scale interlaboratory collaborative studies and incorporates different issues, such as well-appreciated discrepancies in enhancement factor calculations,^35^ and perhaps lesser appreciated problems with characterizing analyte choice,^17^ including size and orientation/steric effects.^42,228^ We note, limit of detection (LoD) may be a convenient way to circumvent problems with EF calculations, however this then brings in instrument-specific effects.

**Fig. 9:**
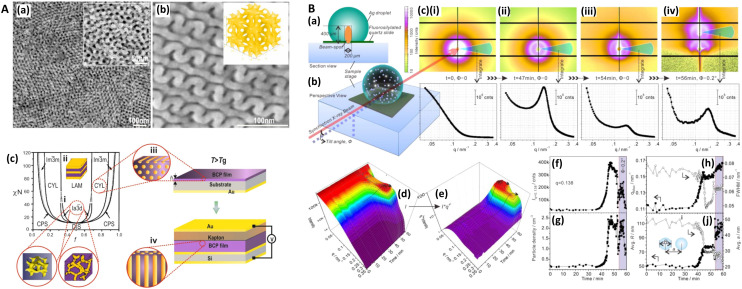
3D SERS. A. Gold-plated three-dimensional nanomorphologies for SERS. Low-angle backscattered scanning electron microscopy (LAB-SEM) images of Au-plated and -sputtered nanostructures of (a) free-standing gyroid, with a unit cell of 20 nm and fill fraction of 21% and (b) double gyroid with the corresponding MATLAB-generated simulations of the nanostructure's cell-unit (inset). (c) Schematic representation of the fabrication of four morphologies for SERS substrates from the available self-assembled range of block co-polymers. (i) Three-dimensional gyroid nanostructures comprised of three rotated arms at the 3-fold junction with each arm attached to another set. (ii) Tuning the volume fractions of the blocks yields lamellae (LAM) and cylinder (CYL) morphologies. (iii and iv) Fabrication of mixed (hexagonal and lying) cylinder arrays and those perpendicular to the substrate (CPS) (iv) *via* annealing of poly- (ferrocenylsilane)-*block*-polylactide film above the glass transition temperature, *T*_g_ in a capacitor like set-up with an applied *E*_f_ of 155 ± 15 V μm^−1^, which are solidified by quenching to room temperature. Mixed morphology comprised of a combination of parallel and perpendicular to the substrate cylinders can be generated in-between (iii) and (iv) by controlling the strength of the applied electric field. Reprinted with permission from Banbury (2019) © American Chemical Society.^79^ B. Synchrotron-radiation small-angle X-ray scattering (SR-SAXS) characterization of 3D SERS matrix. Schematic (a) cross-sectional and (b) perspective views of the experimental setup used for SR-SAXS analysis of a single droplet of a 15 μL Ag sample during the evaporation process, indicating the sample position, the beam-spot size, and the direction of the synchrotron X-ray beam (*λ* = 1.04 Å). (c) Four typical 2D SR-SAXS patterns and their corresponding SR-SAXS curves obtained by integration over an azimuthal range of −165 to 165° recorded at four different times. The tilt angle of the sample stage is 0° in (c)(i)–(iii) and 0.2° in (c)(iv), and the upper SR-SAXS pattern in (c)(iv) originates from light reflection. (d) Time-dependent SR-SAXS curves acquired during the evaporation process, plotted as SR-SAXS intensity (*I*) *vs*. scattering-vector modulus (*q*); the dotted arrow indicates the emergence and rightward shift of the *I* peak. (e) The transformed SR-SAXS curves in (d), plotted as *q*^4^ × *I vs*. *q*; the two dotted arrows indicate the emergence and rightward shift of the *q*^4^ × *I* peaks. Time-dependent (f) *I* values at *q* = 0.138, (g) particle density, (h) peak position (*q*_MAX_, solid squares) and full width at half-maximum (fwhm, hollow squares), and (j) mean sphere diameter (*R*, solid circles) and normalized center-to-center distance (*a*, hollow circles), all of which were derived from the SR-SAXS data presented in (d) and (e). Scattering vector, *q* = (4π(sin *θ*)/*λ*) where *θ* is the scattering angle and *λ* radiation wavelength. Reprinted with permission from Liu (2014) © American Chemical Society.^82^

Fill factors, or the number of nanostructures within a laser-illuminated area, have not been covered, but are important for reasons of areal enhancement, potential electromagnetic near-field interactions (as in Fig. 8A),^104^ and impact on surface wetting. In a HSD SERS substrate context, areal enhancement can also be considered, but the accurate evaluation of the increase in surface area due to nanometric surface asperity is difficult. The lifetime of SERS substrates is important^34^ and substrates’ longevity should be monitored, which may differ significantly for analytical and HSD SERS platforms. Moreover, genuine 3D SERS substrates have not been thoroughly discussed and are arguably their own substrate class altogether (Fig. 9). SERS dependence on impinging radiation angle, as in the magnetically induced orientation effects in plasmonic nanocrescents in Liu (2005) *et al.* (Fig. 8C),^63^ has not been discussed, and likewise, polarization effects, such as in Liang (2009) *et al.*, who studied silver flower-like nanoparticles for SERS.^229^ We note our review is not intended to be exhaustive: some subsections only offer a snapshot. This is especially true for the larger research domains, such as TERS and droplet surface wetting behavior, which have become their own research areas. We have not commented on numerical methods and SERS, or mathematical techniques otherwise employed in the field. This is an important space in SERS where researchers seek to evaluate the sensitivity of SERS media with fundamental theory and simulations. Alongside this, is the exciting cross-over of SERS and machine learning. This emerging area has seen an explosion in interest and would require separate reviews of its own. A Web of Science search reveals that over 60% of ‘SERS’ and ‘machine learning’ research articles were published in 2022 and 2023. The potential of these techniques for characterization of SERS substrates is also summarized in Table 1 alongside the experimental approaches.

Further, we have not remarked on the quality of fabrication of SERS substrates, where plasmonic films could be improved through following some straightforward metallization guidelines,^230^ nor is comment made on temperature effects in SERS and how this can impact on different types of SERS substrates. For instance, Bouillard (2012) *et al.* reported on low-temperature effects in plasmonic nanostructures, which can be used to control electronic and phononic energy distributions, subsequent energy relaxation pathways affecting system ohmic loss, and thus the quality factor of plasmon-polariton resonances.^231^

The distinction between analytical and HSD substrates is not well-defined. Most top-down fabricated, highly ordered geometries are unlikely to be perfectly smooth, whether induced by the nanostructure formation process or by metallization dynamics, and such surface roughness may be substantial enough to support nanolocalized surface plasmon-polaritons. Control of suitably small gaps in lithographically designed SERS substrates is still tricky, and minor spatial variations can disproportionately affect electric field confinement and subsequent SERS. Similarly, not all hotspot-dominated SERS platforms are devoid of order; many might be labeled ‘stochastic’ or quasi-ordered, having structures that exist within a well-defined range of parameters at a specific surface density.^232^

## 8. Conclusions

In this review, we have outlined some of the most important SERS nanosubstrate characterization methods, which standalone in their importance. This overview thus, serves as a catalyst for SERS researchers to explore this emerging and expanding field further with several highlighted novel approaches including for instance, the potential of machine learning, which is still emerging in the nanophotonics, nanoplasmonics and SERS communities, whereas other techniques, such as SERES, are perhaps best described as underappreciated in their potential utility.

Rather than a stark division, all SERS nanosubstrates are on a spectrum where some confine the electromagnetic energy to smaller gaps and provide larger enhancements at the cost of uniformity and reproducibility *i.e.*, control over these small surface nanofeatures, whereas others opt for more reproducible structures on a larger scale (10–100 nm) yet with reduced sensitivity. This trade-off is the essence of the ‘SERS Uncertainty Principle’, introduced by Michael Natan, and to many, it remains an impasse to highly sensitive, highly reproducible SERS nanosubstrates. Others suggest high nanofeature reproducibility may not be necessary, either because only qualitative analysis is required, or because an internal standard can be used to achieve quantification.

Despite differences in SERS nanosubstrates, characterization techniques are often still applied without due consideration as to their utility. SERS has become a sprawling field encompassing many different aspects of science, and in recent years, also increasingly accessible, portrayed by the continued proliferation of literature, within different areas.^16^ Thus, for some, the characterization methods employed may be simply as the result of what is available. Nonetheless, it is essential that SERS nanosubstrates are analysed thoughtfully, meaning that suitable nanocharacterization techniques are applied, and in order to do so, the nature of the nanosubstrate should be carefully considered.

The insights obtained through this review provide an important steppingstone towards a widespread use of high-throughput structured nano-platforms for a variety of applications in nanotechnology and related fields including the development of new nanophotonic devices and the versatility of SERS platforms. This in turn, will make it further applicable in many nano-sensing areas and accelerate the successful nanofabrication of advanced sub-micron platforms, nanophotonic devices with controllable pattern parameters as well as tunable nanoarchitectures with novel properties and applications in nano-optics, nanosensors and nanoelectronics, to name a few.

## Conflicts of interest

The authors declare no conflict of interest.

## Supplementary Material
